# A role for non-coding *Tsix *transcription in partitioning chromatin domains within the mouse X-inactivation centre

**DOI:** 10.1186/1756-8935-2-8

**Published:** 2009-07-20

**Authors:** Pablo Navarro, Sophie Chantalat, Mario Foglio, Corinne Chureau, Sébastien Vigneau, Philippe Clerc, Philip Avner, Claire Rougeulle

**Affiliations:** 1 Unité de Génétique Moléculaire Murine, URA 2578, Institut Pasteur 75724, Paris Cedex 15, France; 2 CEA/Institut de Génomique/Centre National de Génotypage, 2 rue Gaston Crémieux, 91057, Evry Cedex, France; 3 Department of Cell and Developmental Biology, University of Pennsylvania School of Medicine, Philadelphia, PA 19104, USA; 4 UMR 7216 Epigenetics and Cell Fate, Université Paris-Diderot Paris 7, CNRS, 35 rue Hélène Brion 75205 Paris Cedex 13, France

## Abstract

**Background:**

Delimiting distinct chromatin domains is essential for temporal and spatial regulation of gene expression. Within the X-inactivation centre region (*Xic*), the *Xist *locus, which triggers X-inactivation, is juxtaposed to a large domain of H3K27 trimethylation (H3K27me3).

**Results:**

We describe here that developmentally regulated transcription of *Tsix*, a crucial non-coding antisense to *Xist*, is required to block the spreading of the H3K27me3 domain to the adjacent H3K4me2-rich *Xist *region. Analyses of a series of distinct *Tsix *mutations suggest that the underlying mechanism involves the RNA Polymerase II accumulating at the *Tsix *3'-end. Furthermore, we report additional unexpected long-range effects of *Tsix *on the distal sub-region of the *Xic*, involved in *Xic*-*Xic *trans-interactions.

**Conclusion:**

These data point toward a role for transcription of non-coding RNAs as a developmental strategy for the establishment of functionally distinct domains within the mammalian genome.

## Background

The packaging of DNA into a chromatin structure consisting of repeating nucleosomes formed by 146 base pairs of DNA wrapped around an octamer of the four core histones (H2A, H2B, H3 and H4) has been revealed as an incredible source of complexity, allowing the precise control of all biological processes centred on DNA such as transcription, replication, repair and recombination. The amino-terminal domain of the histones is the target of several post-translational modifications underlying the complex relationships between chromatin structure and function [1]. Among them, methylation of lysines 4 and 27 of histone H3 (H3K4 and H3K27, respectively) have been extensively studied at the level of specific loci, as well as chromosome- and genome-wide [2], revealing an accurate correlation between H3K4 methylation and activation or potentiality for transcription on the one hand, and H3K27 methylation and transcriptional repression on the other. These apparently opposite histone modifications can be found either as sharply localized peaks (for example, around promoters) or as more extended domains structuring the genome into 'open' regions of activation or competence (euchromatin), and 'close' regions of long-term repression (heterochromatin). In addition, in pluripotent cells such as embryonic stem (ES) cells, highly conserved non-coding elements and developmentally regulated genes have been shown to be characterized by a combination of methylation at both H3K4 and H3K27, referred to as 'bivalent' domains [3], which allows the establishment of active transcription or long-term silencing upon differentiation. Several large descriptive analyses have been performed to establish correlations between specific histone marks and activation or repression of gene expression. A recent study has reported a striking negative correlation between transcription and H3K27me3, where large domains of H3K27me3 were found to be flanked by expressed genes [4]. However, the molecular mechanisms underlying such a partitioning (and more generally the actual relationship between transcription and chromatin states) remain elusive, especially in mammals.

In this study, we have used the mouse X-inactivation centre (*Xic*), a complex locus responsible for initiation of X-inactivation in mammalian female cells [5], to examine the complex relationships between transcription and H3K4 and H3K27 methylation in ES cells, a model system that recapitulates X-inactivation upon differentiation.

Defined on the basis of chromosomal rearrangements and transgenic studies, the *Xic *contains numerous protein-coding genes such as *Xpct*, *Cnbp2*, *Tsx*, *Cdx4 *and *Chic1*, in addition to the non-coding genes *Ftx*, *Jpx*, *Xist *and *Tsix*. So far, only two of these genes, *Xist *and its antisense partner *Tsix*, have been directly implicated in the regulation of X-inactivation [6]. The *Xist *gene produces a long non-coding RNA that coats the X chromosome in *cis *and is responsible for X-linked gene silencing and X-chromosome-wide acquisition of facultative heterochromatic properties [7]. Controlling the production of increased quantities of *Xist *RNA molecules is, therefore, an essential event in the complex regulation that leads to the inactivation of a single X chromosome in females and the absence of X-inactivation in males. It has been proposed that *Xist *expression shifts from low to high levels at the onset of X-inactivation through the active recruitment of the transcriptional machinery to the *Xist *promoter [8,9].

*Tsix*, which blocks *Xist *RNA accumulation in *cis*, is highly expressed prior to the onset of random X-inactivation, as in undifferentiated ES cells [10]. Using *Tsix *mutations generated in ES cells, it has been demonstrated that *Tsix *expression is required to maintain *Xist *silencing in differentiating male ES cells [11,12], and to ensure a random choice of the X chromosome that will upregulate *Xist *transcription and be inactivated in females [13,14]. The molecular mechanism of the *Tsix*-dependent regulation of *Xist *expression in ES cells has been linked to complex chromatin remodelling activities [8,9,15-17]. In particular, *Tsix *transcription is responsible for the deposition of H3K4me2 along *Xist *[9], except at the *Xist *promoter region, whose euchromatinization is blocked by *Tsix*-induced methylation of both CpG dinucleotides and H3K9 [8].

Intriguingly, the *Tsix *locus is juxtaposed in its 3'-end to a large domain spanning over 340 kb that displays, prior to inactivation, some heterochromatic features of the inactive X. Initially described as a region characterized by H3K4 hypomethylation and H3K9 di-methylation [18], it was then demonstrated that H3K27me3 was also present at the hotspot region [19]. Importantly, both repressive histone marks were shown to be differentially regulated as only H3K9me2 was affected upon loss of the G9a histone methyltransferase [19]. Given the capacity of heterochromatic histone marks to spread across adjacent regions, and the role of histone modifications in the establishment of the appropriate transcriptional activity of *Xist*, an important question that arises concerns the mechanisms that protect the *Xist *locus from heterochromatin spreading from the so-called hotspot region. Here, we demonstrate that *Tsix *transcription is required in *cis *to block H3K27me3 at *Xist*. Based on a series of *Tsix *mutations, we further hypothesize that *Tsix *transcription blocks H3K27me3 spreading from the hotspot, and that the chromatin boundary activity of *Tsix *likely involves the RNA Polymerase II (RNAPII) accumulating at the *Tsix *3'-end. In addition, we show that *Tsix *impacts levels of both H3K27 methylation and gene expression within the hotspot itself, including the *Xpct *gene, which maps to a crucial region that mediates X-chromosome pairing in female ES cells. Our study thus sheds light on the regulation of *Xic *chromatin by *Tsix*, and on the mechanisms delimiting chromatin domains through non-coding transcription in mammalian cells.

## Results

### Distribution of H3K27me3 within the Xic in undifferentiated embryonic stem cells is controlled by Tsix

**Figure 1:**
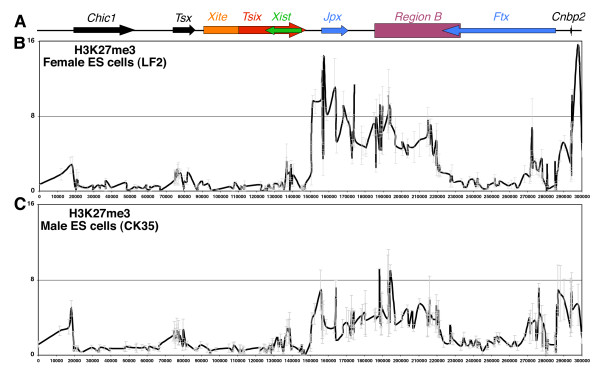
**Distribution of H3K27 tri-methylation within a 300-kb region of the *Xic *in undifferentiated male and female embryonic stem (ES) cells**. **(A) **Schematic diagram of the 300-kb region analyzed in our ChIP experiments showing the location of the different transcription units of the *Xic*. Arrows indicate the direction of each transcription unit. Coding genes are indicated in black, non-coding genes with coloured arrows. The orange box represents the *Xite *locus, an enhancer region that displays transcriptional activity on the major *Tsix *promoter. The purple box represents the so-called region B, a complex transcriptional unit that produces both sense and antisense transcripts [40]. We used in each ChIP experiment a set of 383 primer pairs that were designed automatically to cover the 300-kb region (except repeat regions: see Materials and methods). **(B) **ChIP analysis of H3K27 trimethylation in undifferentiated female (LF2) ES cells and **(C) **in male (CK35 cell line) ES cells. Both graphs show the percentage of immunoprecipitation (%IP) obtained after normalization to the input. All values are mean ± standard deviation. The average percentage of immunoprecipitation calculated for each position was plotted against the genomic location (bp). The +1 coordinate corresponds to position 1,005,322,247 in NCBI build 37.

We have previously identified a large hotspot of H3K27me3 lying 5' to *Xist *in undifferentiated ES cells [18,19]. In order to precisely map the extent and boundaries of the hotspot, we used 383 primer pairs designed across a 300 kb region spanning *Xist*/*Tsix *(Figure 1A) in chromatin immunoprecipitation (ChIP) assays. We found that the H3K27me3 domain is identically structured in several sub-regions in female (Figure 1B) and male (Figure 1C) ES cells, although levels of enrichment were, on average, two- to three-fold higher in females. Whether this difference is sex-linked or based on sex-independent variations between ES cell lines remains to be investigated. Strikingly, a large and predominant region of H3K27me3 is located between the 3'-ends of *Tsix *and *Ftx*, suggesting that H3K27me3 in that region is constrained by the transcription of these two non-coding genes. This is reminiscent to the large BLOCs of H3K27me3 described across the mouse chromosome 17, which are flanked by active genes [4]. Accumulation of H3K27me3 resumes in the 5' region of *Ftx *(with the exception of the *Ftx *promoter; Figure 1) and, as previously shown [19], extends toward *Cnbp2 *and *Xpct *(Supplementary Figure 1), a region required for X-chromosome pairing in female cells [20]. The distribution of H3K27me3 within the *Xic *in undifferentiated ES cells, in particular the inverse correlation observed between H3K27 methylation levels and *Tsix *transcription, suggests that *Tsix *may be involved in constraining H3K27me3 to its 3'-end. This is in agreement with previous data showing increased accumulation of H3K27me3 within *Xist *in the absence of *Tsix *[8,16,17]. To probe this hypothesis, we turned our attention to the analysis of two male ES cell lines in which *Tsix *transcription has been eliminated before reaching *Xist *or drastically reduced: Ma2L, generated through the insertion of a loxP-flanked transcriptional STOP signal within *Tsix *[14], and ΔPas34, in which a potent enhancer of *Tsix*, DXPas34, has been deleted [12].

**Figure 2:**
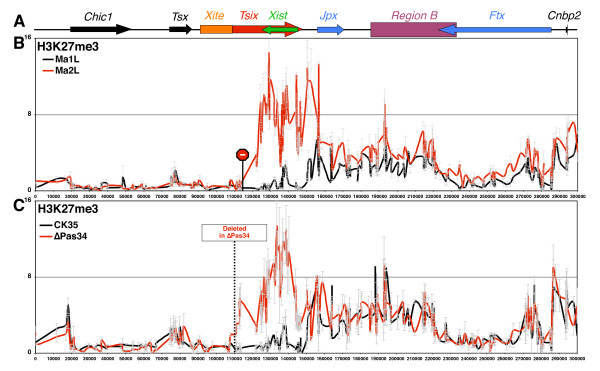
**H3K27 tri-methylation spreads from the hotspot into *Xist *in the absence of *Tsix *transcription**. **(A) **Map of *Xic *(see Figure 1A). **(B) **ChIP analysis of H3K27 trimethylation in male *Tsix*-truncated embryonic stem (ES) cells (Ma2L cell line, red line) and in the corresponding revertant (Ma1L, black line). In the Ma2L cell line, *Tsix *transcription has been truncated through the insertion of a loxP-flanked transcriptional STOP signal downstream of the *Tsix *promoter (represented in the graph by the STOP symbol). The Ma1L revertant was obtained from Ma2L after deletion of the STOP signal. **(C) **Similar analysis in male wild-type (CK35 cell line, black line) and mutated (ΔPas34, red line) ES cells in which *Tsix *transcription is drastically reduced. The ΔPas34 cell line was generated by deleting DXPas34, an enhancer of *Tsix *located near the *Tsix *major promoter. Dotted lines in the graph indicate the location of the 1.2-kb deletion carried by ΔPas34 ES cells.

In the two mutants tested, high levels of H3K27me3 were observed within *Xist *in perfect continuity from the hotspot region (Figure 2). In *Tsix*-truncated cells, the H3K27me3 domain extends up to the inserted transcriptional STOP signal (Figure 2B), and in ΔPas34 cells up to the *Tsix *promoter itself (Figure 2C). Thus, the enrichment for H3K27me3 following *Tsix *invalidation progresses from the *Tsix *3'-end towards the *Tsix *5'-end, and extends beyond *Xist*, indicating a lack of sequence specificity. This apparent sequence-independent, progressive and directional enrichment along the *Xist*/*Tsix *region indicates that, in the absence of *Tsix *transcription, the hotspot of H3K27me3 spreads into the *Xist*/*Tsix *locus and, therefore, suggests that *Tsix *acts as a boundary element partitioning two distinct chromatin domains. More strikingly, when the loxP-flanked transcriptional STOP signal was removed from Ma2L, and *Tsix *transcription restored (Ma1L) [9,14], the boundary of the hotspot domain was re-established at its wild-type location (Figure 2B), which corresponds to the endogenous 3'-end of *Tsix*.

### Xic-wide loss of H3K27me3 during differentiation

**Figure 3:**
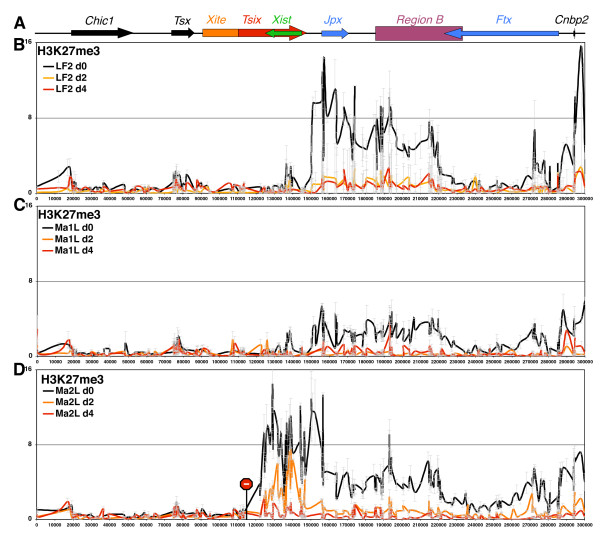
**Loss of H3K27 tri-methylation at the *Xic *during embryonic stem (ES) cell differentiation**. **(A) **Map of *Xic *(see Figure 1A). **(B-D) **Extensive ChIP analysis of H3K27 trimethylation in (B) wild-type female ES cells (LF2), (C) wild-type male revertant cells (Ma1L), or (D) *Tsix*-truncated ES cells (Ma2L). ChIP experiments were performed on undifferentiated ES cells (black line) and after 2 and 4 days (orange and red lines, respectively) of differentiation with retinoic acid.

We then established the profile of H3K27me3 at the *Xic *in differentiating ES cells, when *Tsix *silencing occurs spontaneously [10]. Since *Tsix *blocks H3K27me3 encroachment at *Xist *before differentiation, we expected to find higher levels of methylation at *Xist *following differentiation. However, no significant H3K27me3 enrichment was observed within *Xist *after 2 and 4 days of retinoic acid-mediated differentiation, whether in female (Figure 3B) or male cells (Figure 3C). Instead, a dramatic loss of H3K27me3 within the hotspot region occurs during the first 2 days of differentiation (Figure 3B, C). The entire *Xic *region, including *Xist*, is therefore devoid of H3K27me3 during the time window corresponding to the initiation of X-inactivation in female wild-type ES cells. This result rules out a determinant role for H3K27me3 at either the future active X or the inactive X in the establishment of the appropriate *Xist *expression patterns leading to random X-inactivation. In differentiating *Tsix*-truncated cells, however, significant levels of H3K27me3 can still be detected at *Xist *at day 2 of differentiation, although it becomes undetectable within the hotspot itself (Figure 3D). While this could be attributable to the *Xist *region being more resistant to the loss of ectopically acquired H3K27me3, it reveals that *Xist *is not particularly refractory to H3K27 methylation on differentiation. Thus, the absence of H3K27me3 enrichment at the *Xist/Tsix *region in differentiating wild-type cells is not related to specific regulations of that region that impede H3K27 methylation.

In summary, if *Tsix *were acting by repressing freely diffusing methylation activities acting on *Xist *independently from the hotspot chromatin, the differentiation process and the consequent silencing of *Tsix *should be accompanied by H3K27me3 encroachment at *Xist*. Thus, and given that H3K27me3 at the hotspot region is specific to undifferentiated ES cells, and that the absence of *Tsix *induces H3K27me3 at *Xist *only in undifferentiated ES cells, we propose that the enrichment for H3K27me3 at *Xist *observed in *Tsix*-mutant undifferentiated cells depends on the presence of H3K27me3 at the region upstream of *Xist*, and is related to a deregulation of the hotspot chromatin boundary resulting from the lack of *Tsix *transcription.

### Tsix-mediated H3K4 methylation is not involved in blocking H3K27me3 spreading

One hypothesis of how *Tsix *might block the spreading of H3K27me3 is related to the chromatin remodelling activities that *Tsix *displays at the *Xist *locus. We have indeed previously reported that *Tsix *transcription triggers the deposition of H3K4me2 across its own transcription unit [9]. In addition, *Tsix *is responsible for establishing a repressive chromatin structure over the *Xist *promoter, characterized by elevated levels of H3K9 and DNA methylation and by low levels of H3K4 methylation and H3K9 acetylation [8].

**Figure 4:**
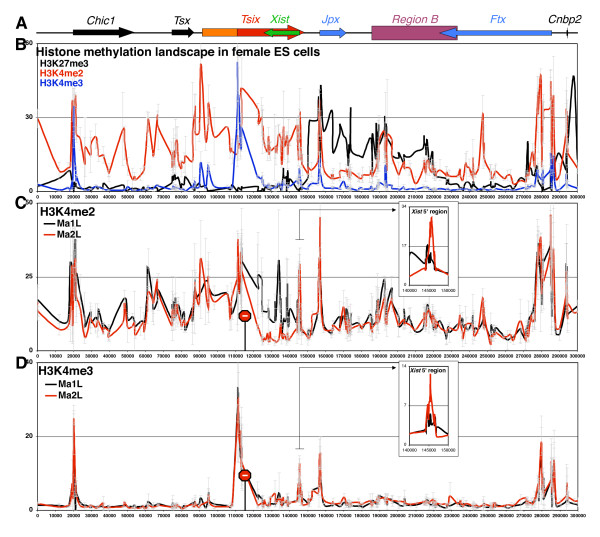
**H3K4 methylation is not involved in the H3K27me3 boundary formation at the *Xic***. **(A) **Map of *Xic *(see Figure 1A). **(B) **ChIP analysis showing the distribution of histone H3 modifications at the *Xic *in female embryonic stem (ES) cells (LF2) using antibodies against H3K27 trimethylation (H3K27me3, black line), H3K4 dimethylation (H3K4me2, red line) and H3K4 trimethylation (H3K4me3, blue line). **(C) **Extensive analysis of H3K4me2 and **(D) **H3K4me3 in wild-type (Ma1L, black line) and *Tsix*-truncated (Ma2L, red line) male ES cells. Insets focus on the percentage of immunoprecipitation observed at the *Xist *5' region.

To probe the relationship between *Tsix *transcription, H3K27me3 and H3K4 methylation, we extended the analysis of H3K4 di- and tri-methylation profiles to the entire *Xic *region. We observed that the *Xic *is structured in two distinct domains: a large domain enriched for H3K4me2 extending from *Chic1 *to the *Tsix *3'-end, juxtaposed to the hotspot of H3K27me3 from the *Tsix *3'-end onwards (Figure 4B). Some interspersed regions of H3K4me2 were found downstream of *Tsix*, such as at the promoters of *Jpx*, *Ftx *and *Cnbp2*. As previously demonstrated [8,9], the lack of *Tsix *transcription was associated with the loss of H3K4me2 across *Tsix *with the exception of the *Xist *promoter itself, where higher levels were found in Ma2L (Figure 4C). It is important to note that the level of background detected with the H3K4me2 antiserum used in this study is relatively high. The residual H3K4me2 signal observed in the H3K27me3 hotspot region and within *Xist *in the *Tsix *mutant cell line indeed corresponds to background, as previously shown using a no-longer available antiserum ([9] and data not shown).

In agreement with genome-wide analyses [2], the profile of H3K4me3 was highly restricted to active promoter regions, such as those of *Chic1*, *Tsix*, *Jpx *and *Ftx*, in both female and male ES cells (Figure 4B, D). Promoters of genes displaying low levels of expression, such as *Cnbp2 *and *Xist*, were not consistently marked with H3K4me3. In addition, some regions such as the promoter of *Jpx *were found enriched in both H3K4me3 and H3K27me3, thus defining a bivalent domain. Truncation of *Tsix *did not affect the levels and distribution of H3K4me3 across the region, with the exception of the *Xist *promoter, which displayed elevated levels in the absence of *Tsix*, as previously reported [8].

The global mirror image between H3K4me2 and H3K27me3 at the *Xic*, together with the opposite influence of *Tsix *on both marks across *Xist*/*Tsix*, could suggest that *Tsix *blocks H3K27me3 spreading through the deposition of H3K4me2. Two observations, however, indicate that this is likely not the case. First, in the absence of *Tsix*, the *Xist *promoter becomes methylated on both K4 and K27 residues of H3 (Figures 3D and 4C). Second, regions enriched for both marks have been reported from both genome-wide analysis [3] and our analysis of the *Xic *(Figure 4). This strongly argues against the idea that the H3K4me2 domain triggered by *Tsix *is a barrier for H3K27 tri-methylation.

### Mechanistic insights into the boundary function of Tsix

The profile of H3K27me3 accumulation in wild-type cells reveals that the boundary of the H3K27me3 hotspot maps to position 150,000, which approximately corresponds to the region of *Tsix *transcription termination as previously described [21]. In order to investigate the molecular basis for the barrier activity of *Tsix*, we analyzed the distribution of RNAPII molecules across the *Xist*/*Tsix *region.

**Figure 5:**
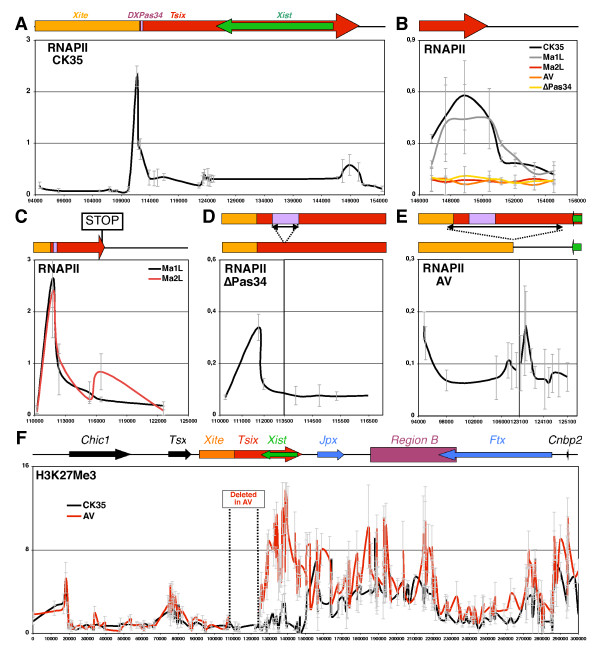
**Systematic accumulation of *Tsix*-associated RNA Polymerase II (RNAPII) at the limit of the H3K27 tri-methylation domain of the *Xic *in undifferentiated embryonic stem (ES) cells**. **(A) **Profile of RNAPII distribution in wild-type male ES cells (CK35 cell line). A schematic representation of the *Xist*/*Tsix *locus is shown (top). Red and green arrows indicate the orientation of *Tsix *and *Xist *transcription units, respectively. The orange box represents the *Xite *locus. The purple box corresponds to the DXPas34 enhancer. **(B) **Distribution of RNAPII at the *Tsix *3' region in wild-type (CK35, in black) and three different *Tsix*-mutant male ES cell lines (Ma2L in red, ΔPas34 in yellow and AV in orange). The RNAPII profile is also shown for the corresponding Ma2L control, Ma1L (in grey). The AV cell line, derived from CK35 wild-type cells, carries a 15-kb-long deletion encompassing the major *Tsix *promoter. **(C-E) **RNAPII accumulation in three independent *Tsix *mutant male ES cells: Ma2L (C), ΔPas34 (D) and AV (E). A schematic representation of the region analyzed in each ChIP assay is shown (top). The colour code of the genetic elements is the same as in (A). In (C), the position of the transcriptional STOP signal introduced in the Ma2L cell line is indicated. In (D, E), arrows indicate position of the deletions introduced to generate ΔPas34 and AV, respectively. **(F) **ChIP analysis of H3K27 tri-methylation in wild-type (CK35, in black) and AV male ES cells (in red). Dotted lines indicate the position of the 15-kb region deleted in the AV cell line.

In wild-type cells, RNAPII was shown to markedly accumulate at the *Tsix *5' region (Figure 5A). Levels of RNAPII then remain low along the *Xist *locus and appear enriched between positions 147,000 and 151,000 (Figure 5A, B), as expected for a region where transcription termination and RNA cleavage of *Tsix *should occur, causing pausing and accumulation of RNAPII molecules. Strikingly, this peak of RNAPII corresponds precisely to the boundary of H3K27me3, as mapped above (around position 150,000). This observation suggests that the accumulation of RNAPII molecules itself at the *Tsix *3'-end could be involved in blocking the spreading of H3K27me3 from the hotspot to within *Xist*/*Tsix*. To probe this hypothesis, we measured RNAPII accumulation across this region in two *Tsix *mutant cell lines (Ma2L and ΔPas34) in which the boundary of the hotspot has been displaced. The enrichment for H3K27me3 over this region in the mutants correlates with a complete loss of RNAPII accumulation (Figure 5B). Strikingly, RNAPII was rather found to accumulate at the ectopic boundary of the H3K27me3 domain. This corresponds precisely to the site of ectopic termination of *Tsix *in Ma2L (Figure 5C), and to the *Tsix *promoter in ΔPas34 (Figure 5D), which, although strongly repressed, still recruits significant levels of RNAPII [12]. These observations, made both in wild-type cells and in two independent *Tsix*-mutant ES cell lines, strongly support the hypothesis that RNAPII accumulation is involved in establishing the H3K27me3 boundary. Importantly, when *Tsix *transcription across *Xist *is restored through the deletion of the transcriptional STOP signal of Ma2L to generate Ma1L, the resulting accumulation of RNAPII at the *Tsix *3'-end (Figure 5B) is accompanied by the restoration of the boundary of H3K27me3 at its natural location (Figure 2B).

According to such an hypothesis, mutations deleting the promoter region of *Tsix *should induce the spreading of H3K27me3 up to the next accumulation of RNAPII molecules. To address this, we exploited the male AV cell line, in which 15 kb encompassing the *Tsix *promoter have been deleted, resulting, similarly to ΔPas34 and Ma2L, in a strong reduction of *Tsix *transcription across *Xist *[12], and in the absence of RNAPII accumulation at the endogenous *Tsix *3'-end (Figure 5B). The extent of the deletion carried by AV results in the *Xite *locus, a complex enhancer region with intrinsic non-coding transcriptional activity, acting on the major *Tsix *promoter [22-24], and the minor distal promoter of *Tsix *[21] being brought in the vicinity of the *Xist *3'-end (Figure 5E). Interestingly, we could detect a low but significant accumulation of RNAPII at the junction of the deletion, some 3 kb downstream of the *Xist *3'-end (Figure 5E), which likely results from non-coding transcription events initiating either at *Xite *or at the minor *Tsix *promoter and terminating before reaching *Xist*.

Similarly to Ma2L and ΔPas34, the lack of accumulating RNAPII at the *Tsix *3'-end in AV (Figure 5B) correlates with an extension of the hotspot domain of H3K27me3 to within *Xist *(Figure 5F). Interestingly, upstream of the deletion junction (positions 0 to 107,000), levels of this repressive mark remain similar to that of wild-type cells. Thus, in AV, the novel boundary of the H3K27me3 domain also corresponds to the ectopic accumulation of RNAPII at the shortened region located in between *Xite *and *Xist*. In summary, our results show in five different genetic contexts (wild type, Ma1L, Ma2L, ΔPas34, and AV cells) a perfect correlation between the limit of the H3K27me3 domain and the accumulation of *Tsix*-associated RNAPII. These data strongly suggest that, in wild-type cells, the accumulation of RNAPII at the *Tsix *3'-end forms the molecular basis of the boundary activity of *Tsix*.

### Distal effects of Tsix on Xic chromatin and Cnbp2 and Xpct gene expression

In addition to its effect on the hotspot boundary, changing the extent and/or levels of *Tsix *transcription was found to affect H3K27me3 levels within the hotspot itself (Figures 2 and 5F), with increased levels observed in particular in Ma2L and AV and, to a lesser extent, in ΔPas34. This is in agreement with the hypomorphic nature of the ΔPas34 mutation (in which some 10% of *Tsix *RNA remains expressed) [12], and indicates that 90% reduction of *Tsix *activity is sufficient to deregulate the natural hotspot boundary but not to allow increased enrichment of H3K27me3 at the hotspot itself. The perfect superposition of H3K27me3 levels in the proximal part of the *Xic *(across *Chic1 *and *Tsx*) between control and mutant cells, as well as the absence of changes of H4K4me2 and me3 levels (Figure 4), reinforces the significance of this slight increase in H3K27me3 observed within the hotspot region. Importantly, this increase is also observed at *Xpct*, as confirmed by the analysis of 43 independent primer pairs and the use of an independent anti-H3K27me3 antibody (Additional file 2).

**Figure 6:**
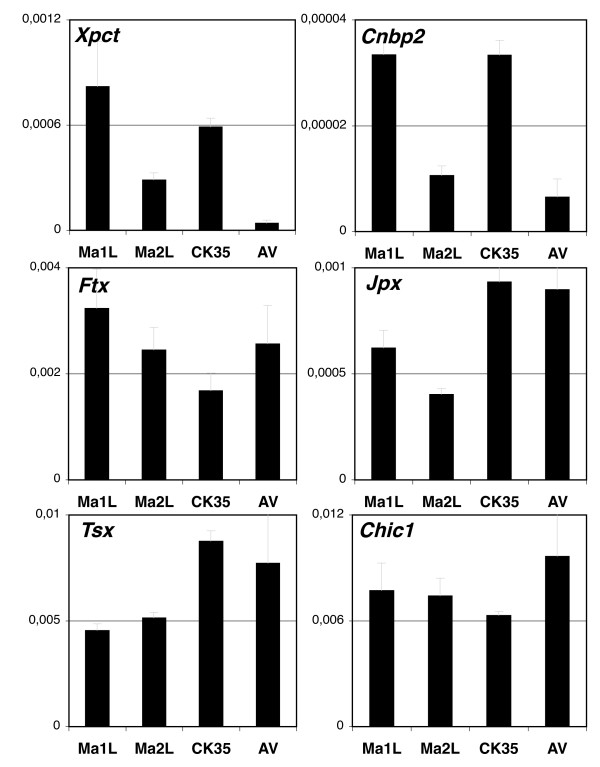
***Tsix *transcription affects the expression of genes located in the distal part of the *Xic***. RT-PCR analysis of several genes lying within the *Xic*: *Xpct*, *Cnbp2*, *Ftx*, *Jpx*, *Tsx *and *Chic1 *in different embryonic stem cells, after normalization to *Arpo PO *transcript levels.

This unexpected long-distance effect of *Tsix *on *Xic *chromatin prompted us to determine the impact of *Tsix *on the expression of genes lying within the *Xic*. The expression of *Xpct*, *Cnbp2*, *Ftx*, *Jpx*, *Tsx *and *Chic1 *was analyzed by real-time RT-PCR in control and two independent *Tsix*-mutant male ES cells (Figure 6). In agreement with the low and *Tsix*-independent levels of H3K27me3 in the proximal part of the *Xic*, expression levels of *Tsx *and *Chic1 *were found to be unaffected by the loss of *Tsix*. In contrast, we detected a marked down-regulation of *Xpct *and *Cnbp2 *transcript levels in both the Ma2L and AV mutants (Figure 6) compared to their respective controls. We conclude that *Tsix *controls tri-methylation of H3K27 across a large region extending over more than 300 kb, from *Xpct *to *Tsix*, and impacts on expression levels of some of the genes located within this region.

## Discussion

In this study, we have used the X-inactivation centre as a model to understand the impact of non-coding transcription in structuring and partitioning chromatin domains within a developmentally regulated region. Focusing in particular on a large heterochromatic-like domain that flanks the *Xist *master locus, this study has revealed new functions for the *Tsix *antisense transcript in controlling levels and distribution of H3K27me3 together with gene expression over an extended sub-region of the *Xic*.

### Long-range control of H3K27me3 levels by non-coding transcripts

The existence of antisense non-coding RNAs responsible for monoallelic gene expression establishment and/or maintenance at both the *Xic *(such as *Tsix*) and autosomal imprinted domains is one of the most striking molecular parallels between X-inactivation regulation and genomic imprinting [25]. Surprisingly, however, although antisense transcripts controlling autosomal imprinting, such as *Air *and *Kcnq1ot1*, have been shown to repress both overlapping and distal genes through methylation of both H3K27 and H3K9 [26-28], existing data concerning *Tsix *favoured a role as a specific regulator of *Xist*. We have now challenged this conclusion by showing that *Tsix *fine-tunes H3K27me3 at the hotspot itself, and is required for appropriate expression of *Cnbp2 *and *Xpct *genes. Interestingly, the modification of H3K27me3 levels did not affect the methylation of H3K4 and H3K9 (Additional file 3). It appears, therefore, that regulation of H3K27me3 is independent of that of H3K9me2 (as shown in G9a-null ES cells [19]), and *vice versa*.

The increase in H3K27me3 levels at the hotspot observed in ES lines harbouring a mutated *Tsix *allele could be explained by the increase of *Xist *RNA that characterizes *Tsix*-null ES cells [11,12], which could lead to *Xist *RNA spreading and silencing in *cis*. Interestingly, the recent demonstration that, in undifferentiated ES cells, both *Xist *and *Tsix *RNAs interact with the polycomb machinery required to tri-methylate H3K27 (PRC2) [29] provides a potential molecular scenario accounting for our results. In this model, both *Xist *and *Tsix *RNAs compete for PRC2, with *Xist *RNA-PRC2 acting as a trigger of methylation at the hotspot region, and *Tsix *RNA acting as a competitor that inhibits the formation or activity of the methylating *Xist *RNA-PRC2 complex. This implies that, when *Xist *RNA-PRC2 interactions overcome that of *Tsix *RNA-PRC2, such as in *Tsix*-mutant cells, the level of methylation within the hotspot increases.

### Complex control of Xist chromatin by Tsix

The molecular mechanism of the *Tsix*-dependent regulation of *Xist *transcription has been linked to complex chromatin modifications of the *Xist *locus. The *Xist *promoter region was shown to be abnormally enriched for euchromatin-associated histone marks and depleted for heterochromatin-associated modifications in *Tsix*-truncated male ES cells [8], a result that we have confirmed in the present study using 383 primer pairs. Similar to what was observed in terminally differentiated cells derived from male embryos carrying a *Tsix*-truncated allele [15], we show in this report that such an accumulation of active chromatin marks is maintained, or even increased, during differentiation of *Tsix*-truncated cells (Additional file 4), correlating with *Xist *transcription upregulation [8,12]. We conclude that *Tsix *controls *Xist *transcription through repression in *cis *of the *Xist *promoter chromatin.

In addition to inducing a repressive chromatin structure at the *Xist *promoter, *Tsix *generates an 'open' chromatin state along *Xist *by triggering H3K4me2 on the one hand [9] and blocking H3K27me3 enrichment on the other [8,16,17]. *Tsix *therefore appears to have dual effects on *Xist *chromatin, 'opening' the chromatin structure along *Xist *but repressing it at the *Xist *promoter itself. What might be the function of the H3K27me3-protective activity of *Tsix*? Since *Tsix*-mutant ES cells devoid of H3K27me3 at *Xist *hyperactivate *Xist *transcription on differentiation [16], it appears that encroachment of this repressive mark renders *Xist *activation less efficient, probably by inhibition of transcription elongation. Interestingly, no sign of H3K27me3 could be detected along *Xist *in early differentiating female or male wild-type ES cells, despite the analysis of 58 positions along *Xist*, including the *Xist *promoter region. Thus, H3K27me3 at *Xist *does not appear to play any role in the normal regulation of *Xist *expression, neither from the active nor the inactive X chromosome. We propose that upon loss of *Tsix *expression during differentiation, the *Xist *promoter chromatin shifts into euchromatin under a global context devoid of H3K27me3. This should favour both the recruitment of the transcriptional machinery at the *Xist *promoter and efficient *Xist *transcription elongation.

Moreover, given that the *Xist*/*Tsix *region is involved in X-chromosome pairing [30,31], and that *Tsix*-mutant cells do not perform this pairing event appropriately [30], we propose that the inhibition of H3K27me3 at *Xist *by *Tsix *is required for the establishment of *Xic*-*Xic *interactions involving the *Xist*/*Tsix *region. This would ensure accurate pairing of the X chromosomes and efficient *Xist *upregulation.

### Tsix transcription as a chromatin barrier element

Our extensive analysis of H3K27me3 within the *Xic *has revealed that a large part of the H3K27me3 hotspot is located between the 3'-ends of two expressed non-coding genes, *Tsix *and *Ftx*, suggesting that transcription of both genes is constraining the H3K27 methylation to the region in between. This is reminiscent of the situation recently described for mouse chromosome 17, where large domains or BLOCs of H3K27me3 are flanked by active genes [4]. Importantly, our ChIP-PCR analysis of the *Xic *is highly similar to that obtained from ChIP-Seq experiments performed by others [32] (Additional file 5).

Based on the localization and characterization of the boundary of the H3K27me3-rich domain in both wild-type and four independent *Tsix*-mutant ES cells, our results strongly indicate that H3K27me3 at *Xist *results from a spreading process initiated at the hotspot. Under this scenario, it is noteworthy that the absence of methylation at *Xist *observed in differentiating cells, when *Tsix *is silenced, correlates with a global reduction of H3K27 tri-methylation within the hotspot. Therefore, we conclude that two mechanisms have evolved in order to prevent *Xist *heterochromatinisation: in undifferentiated ES cells, active *Tsix *transcription constrains the hotspot domain to its 3'-end, whilst in differentiating cells *Tsix *silencing coincides with the loss of H3K27me3 in the hotspot, thus preventing the spreading of such a modification into *Xist*.

It is interesting to note that the boundary of the H3K27me3 domain located at the *Tsix *3'-end appears devoid of H3K4me3 (Figure 4) and H3K9 acetylation (Additional file 6), two marks frequently associated with chromatin boundary elements [33]. In addition, although we have previously shown that the insulator protein CTCF is bound at the *Tsix *3'-end [8], this binding is maintained upon *Tsix *truncation [8], clearly demonstrating that CTCF is not a formal obstacle to the spreading of H3K27 tri-methylation within the *Xic*. Other activities, likely related to transcription, should therefore be important for such barrier activity. In this regard, the systematic accumulation of RNAPII, either ectopic or at the natural end of *Tsix*, strongly suggests that the RNAPII accumulating at the 3'-extremity of *Tsix *provides the molecular basis for the boundary function of *Tsix *transcription. These data are reminiscent of those from other systems where RNA Polymerase III transcription machinery has been shown to delimit chromatin domains. Examples include a tRNA transcription unit that acts as a barrier to the spread of repressive chromatin [34], and inverted repeat boundary elements flanking the fission yeast mating-type heterochromatin domain that have been shown to recruit TFIIIC to block heterochromatin spreading [35]. The high density of RNAPII accumulating within the 4-kb region at the 3'-end of *Tsix *might induce a local gap in the nucleosome array [36] that would block the spread of H3K27me3 into *Xist*. Preliminary studies do not, however, favour this hypothesis, as the distribution of histone H3 is similar across the boundary region and in flanking domains (data not shown). Hence, we speculate here that yet unknown activities associated with the RNAPII complex transcribing *Tsix *are directly responsible for blocking the spread of H3K27me3. One candidate is the UTX H3K27 demethylase, which was shown in *Drosophila *to interact with the elongating form of RNAPII [37]. Altogether, these observations led us to propose that the accumulation of unproductive RNAPII at the 3'-end of *Tsix *functions as a genomic landmark protecting *Xist *from H3K27 tri-methylation. This illustrates the recent idea that barriers to heterochromatin spreading might operate by exploiting the function of other gene-regulatory mechanisms, without involving specialized apparatus dedicated to such function [33]. In agreement with this, developmentally regulated activation of a SINE element in mammals was recently shown to function as a domain boundary [38].

## Conclusion

Given the large extent of developmentally regulated non-coding transcription of unknown function recently discovered in mammalian genomes [39], and the *Xic*-wide chromatin-organizing function of *Tsix *that we report here, it is tempting to speculate that non-coding transcription might generally function as organizer of distinct chromatin domains required to establish appropriate expression patterns of adjacent classical protein-coding genes. Whether coding transcription can play a similar role under specific circumstances, as recently suggested [4], remains to be investigated.

## Materials and methods

### Cell culture

ES cell lines were grown in DMEM, 15% foetal calf serum, and 1,000 U/ml LIF (Chemicon/Millipore, Billerica MA, USA). Female LF2 ES cells were cultured on gelatin-coated plates in the absence of feeder cells. Male Ma1L, Ma2L (a kind gift of R. Jaenisch), CK35, ΔPas34 and AV ES cells were cultured on Mitomycin C-treated male embryonic fibroblast feeder cells, which were removed by adsorption before chromatin and RNA extractions. To induce ES cell differentiation, cell lines were plated on gelatin-coated flasks and cultured in DMEM, 10% foetal calf serum, supplemented with retinoic acid at a final concentration of 10^-7 ^M. The medium was changed daily throughout differentiation. Retinoic acid-treated ES cell lines exhibited morphological features of differentiated cells. Differentiation was also evaluated by Oct3/4 and Nanog expression analysis by real-time PCR (data not shown).

### Chromatin immunoprecipitation

ChIP assays were performed as previously described [8]. The antibodies RNAPII (Euromedex, Souffelweyersheim, France), H3K4me2, H3K9me3, H3K9Ac, and H3K27me3 (Upstate Biotechnology/Millipore, Billerica MA, USA) and H3K4me3 and H3K27me3 (Abcam, Cambridge, UK) were used at 1:500, 1:100, 1:100, 1:100, 1:500, 1:250 and 1:100 dilutions, respectively, to immunoprecipitate an equivalent 20 μg of DNA in ChIP assays. Each assay was performed two to six times on independent chromatin preparations to control for sample variation. To standardize between experiments, we calculated the percentage of immunoprecipitation by dividing the value of the IP by the value of the corresponding input, both values first being normalized for dilution factors.

### Real-time PCR analysis of ChIP assays

To analyze ChIP experiments, real-time PCR assays were performed in 384-well plates. The primers (Additional file 7) used for the ChIP assays were designed automatically to produce 90- to 140-base-pair amplicons that cover the *Xic *region spanning from position 100,532,247 to 100,832,343 on NCBI build 37. A program was written with the aim of producing high quality primers and maximizing coverage in this region (available upon request). The program accesses our local mouse genome database in order to extract the sequence designated as a template, and to mask all sequence variations and repeats based on the NCBI mouse genome build release 37. This template is passed to Primer 3 with the following minimum, optimum, and maximum values applied: GC contents of 30%, 50% and 80%; and Tm of 58°C, 60°C, and 61°C. The resulting primers are further analyzed by sequence homology using BLAST and the mouse genome as the query sequence to eliminate those that can produce more than one PCR product, and to filter out those where the log base 10 of significant hits per pair exceeds 2. Besides the GC content and Tm values already mentioned, our program also screens for long runs of identical nucleotides, and G/C stretches at the 3'-ends. For all primer pairs, PCR efficiencies were verified to be similar.

In addition to the automatic primer selection strategy, liquid handling of the 384-well plate was performed with a Baseplate robotic workstation (The Automation Partnership, Hertfordshire, UK). The composition of the quantitative PCR assay included 2.5 μl DNA (the immunoprecipitated DNA or the corresponding input DNA), 0.5 μM forward and reverse primers, and 1X Power SYBR^® ^Green PCR Master Mix (Applied Biosystems, Foster City CA, USA). The amplifications were performed as follows: 2 minutes at 95°C, 40 cycles at 95°C for 15 s and 60°C for 60 s in the ABI/Prism 7900HT real-time PCR machine (Applied Biosystems). The real-time fluorescent data from quantitative PCR were analyzed with the Sequence Detection System 2.3 (Applied Biosystems).

### Quantitative RT-PCR

Random-primed RT was performed at 42°C with Superscript II reverse transcriptase (Invitrogen) with 4 μg of total RNA isolated from cell cultures with RNable (Eurobio, Les Ulis, France). Control reactions lacking enzyme were verified negative. Quantitative real-time PCR measurements using SYBR Green Universal Mix were performed in duplicates, and *Arpo P0 *transcript levels were used to normalize between samples.

## Abbreviations

ChIP: chromatin immunoprecipitation; ES: embryonic stem; RNAPII: RNA Polymerase II; *Xic*: X-inactivation centre.

## Competing interests

The authors declare that they have no competing interests.

## Authors' contributions

PN conceived of the study, designed, carried out and analyzed the experiments, and wrote the manuscript. SC carried out and analyzed the experiments, and co-wrote the manuscript. MF carried out the bioinformatic studies for designing the ChIP primer pairs, CC and SV helped to carry out the experiments, PC participated in drafting the manuscript, and PA and CR conceived of the study and co-wrote the manuscript. All authors read and approved the final manuscript.

## Supplementary Material

Additional file 1 **Figure S1**. Enrichment for H3K27 tri-methylation extends towards *Cnbp2 *and *Xpct *genes. The diagram at the top represents the 500-kb region analyzed in our ChIP experiments with the primer pairs used (P1 to p43). For legends, see Figure 1A. The open blue box indicates the location of the *Tsix *3' region (covered by primers p20 to p31). Note that we used for this figure different sets of primer pairs and a different anti-H3K27me3 antibody (Upstate/Millipore, Billerica MA, USA) than those used for Figures 1 to 5 (Abcam). Click here for file

Additional file 2 **Figure S2**. Increased accumulation of H3K27 tri-methylation in the *Cnbp2*-*Xpct *region is observed in different *Tsix *mutant ES cells. (A) The diagram at the top is the same as in Additional file 1. (B-D) The graphs show the percentage of immunoprecipitation obtained with H3K27me3 antibodies (Upstate) at 43 different positions across the *Xic *in different mutants (in red) and their corresponding control cell lines (in black): (B) Ma1L/Ma2L, (C) CK35/ΔPas34, and (D) CK35/AV. Click here for file

Additional file 3 **Figure S3**. *Tsix *does not affect H3K9 di-methylation within the *Xic*. ChIP analysis of H3K9 di-methylation in *Tsix*-truncated (Ma2L, in red) and corresponding control (Ma1L, in black) embryonic stem cell lines. Locations of the primer pairs are depicted in Additional file 1. Click here for file

Additional file 4 **Figure S4**. The *Xist *promoter region is abnormally enriched in euchromatic marks in *Tsix*-truncated undifferentiated and differentiating embryonic stem cells. ChIP analysis of H3 modifications around the *Xist *5' region in *Tsix*-truncated cells (Ma2L) and its corresponding control (Ma1L) male ES cells. (A) H3K4 dimethylation (H3K4me2), (B) H3K4 trimethylation (H3K4me3), (C) H3K9 acetylation (H3K9Ac) and (D) H3K9 trimethylation (H3K9me3). ChIP experiments were performed on undifferentiated embryonic stem cells (d0; black and orange lines for Ma1L and Ma2L, respectively) and after 4 days of differentiation with retinoic acid (d4; blue and red lines for Ma1L and Ma2L, respectively). Click here for file

Additional file 5 **Figure S5**. Comparison of ChIP-PCR and available ChIP-Seq data. A schematic representation of the 300-kb region analyzed in our ChIP experiments is shown at the top. Bottom: results obtained for H3K4me2, H3K4me3 and H3K27me3 from our ChIP-PCR (graphs in black) experiments and from [32] (in colour). Click here for file

Additional file 6 **Figure S6**. H3K9 acetylation does not mark the boundary of the H3K27 tri-methylated domain located at the *Tsix *3' end. A schematic representation of the 300-kb region analyzed in our ChIP experiments is shown at the top. For legends, see Figure 1A. ChIP analysis of H3K27me3 (in black) and H3K9 acetylation (H3K9Ac, in red) in wild-type male embryonic stem cells (Ma1L). ChIP assays were performed using the set of 383 primers pairs. Click here for file

Additional file 7 **Figure S7**. Primers used in the 384-well PCR plate-based analysis. Click here for file
