# Supplementary figures and images for: A role for non-coding Tsix transcription in partitioning chromatin domains within the mouse X-inactivation centre
Source: Epigenetics Chromatin. 2009 Jul 20;2:8. doi: 10.1186/1756-8935-2-8 (PMC2720958; doi:10.1186/1756-8935-2-8)

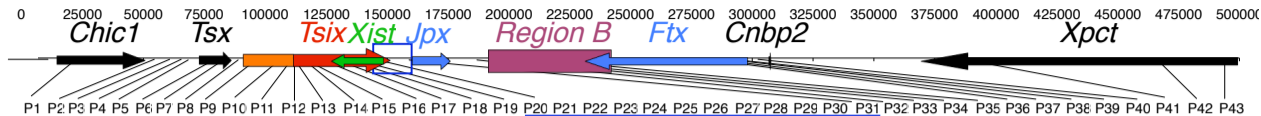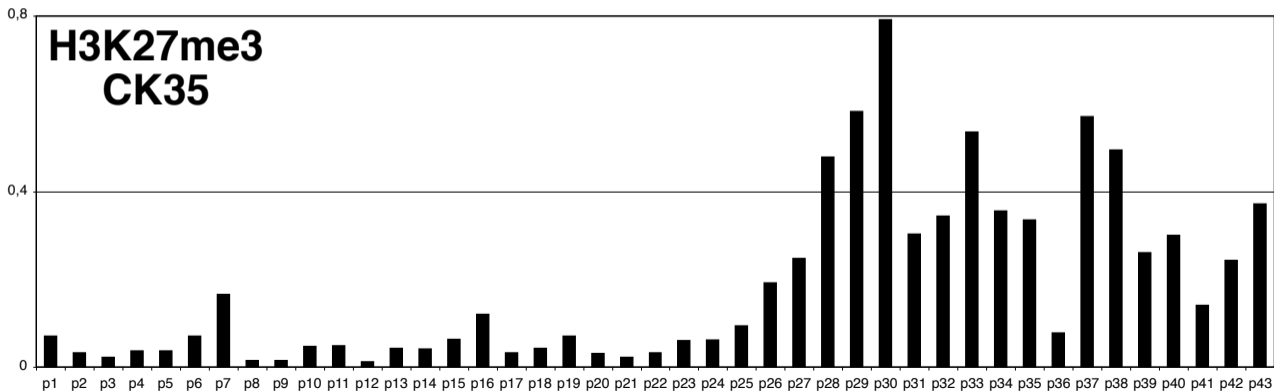

Supplement: Additional file 1 — Figure S1. Enrichment for H3K27 tri-methylation extends towards Cnbp2 and Xpct genes. The diagram at the top represents the 500-kb region analyzed in our ChIP experiments with the primer pairs used (P1 to p43). For legends, see Figure 1A. The open blue box indicates the location of the Tsix 3' region (covered by primers p20 to p31). Note that we used for this figure different sets of primer pairs and a different anti-H3K27me3 antibody (Upstate/Millipore, Billerica MA, USA) than those used for Figures 1 to 5 (Abcam). [file 1756-8935-2-8-S1.pdf]

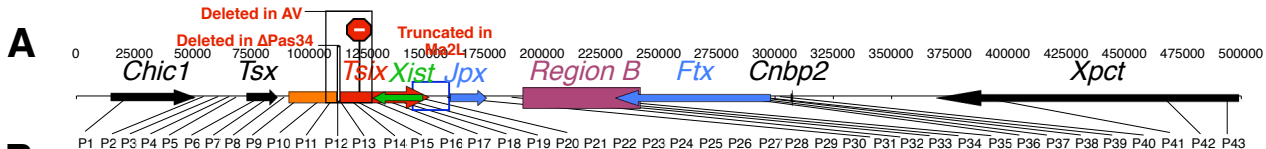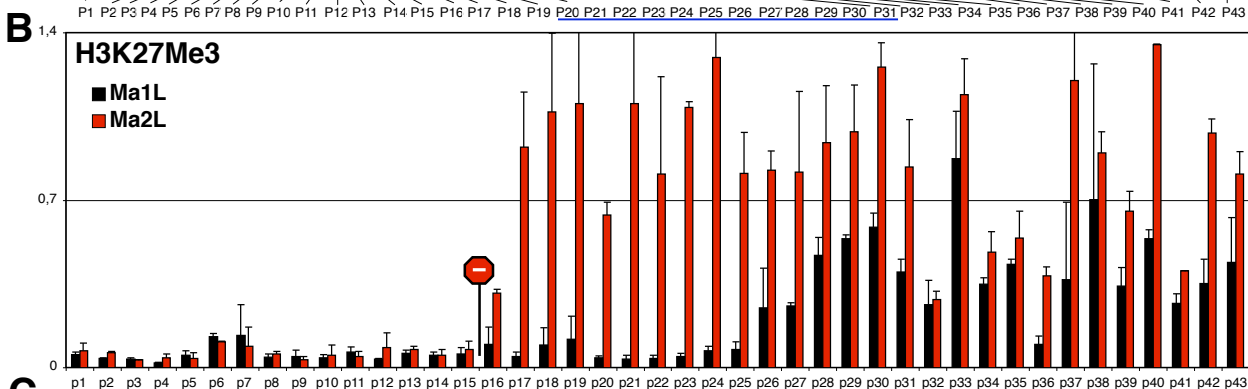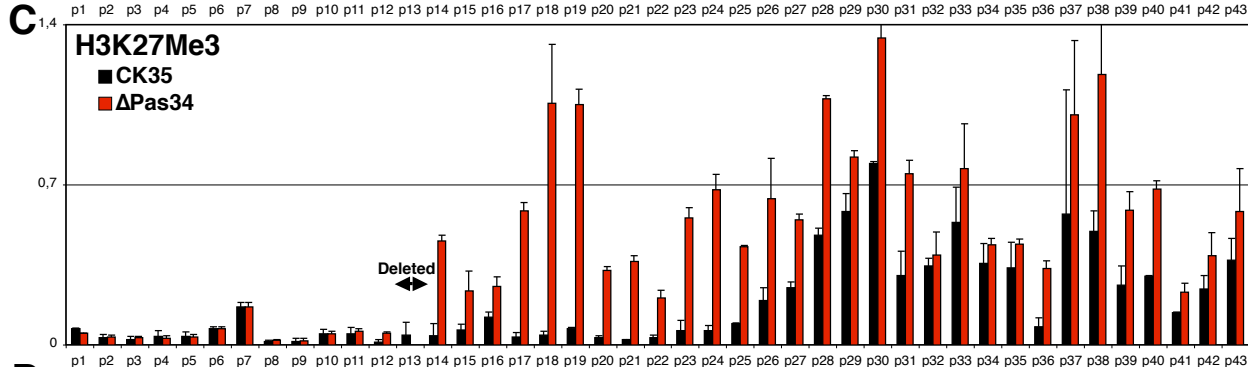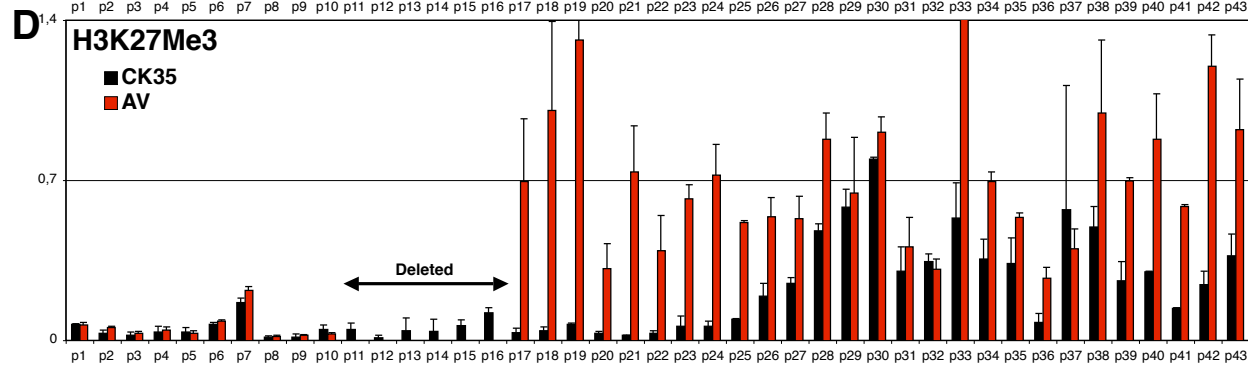

Supplement: Additional file 2 — Figure S2. Increased accumulation of H3K27 tri-methylation in the Cnbp2-Xpct region is observed in different Tsix mutant ES cells. (A) The diagram at the top is the same as in Additional file 1. (B-D) The graphs show the percentage of immunoprecipitation obtained with H3K27me3 antibodies (Upstate) at 43 different positions across the Xic in different mutants (in red) and their corresponding control cell lines (in black): (B) Ma1L/Ma2L, (C) CK35/ΔPas34, and (D) CK35/AV. [file 1756-8935-2-8-S2.pdf]

# H3K9Me2

■ Ma1L  
■ Ma2L

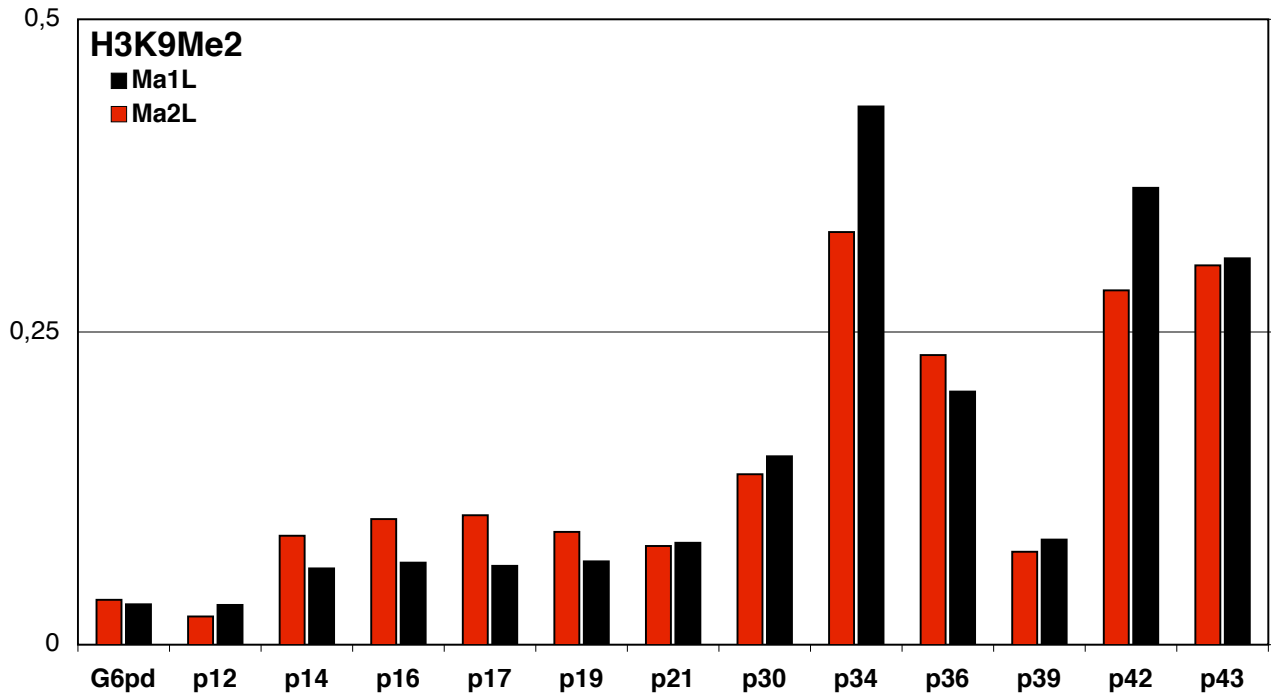

Supplement: Additional file 3 — Figure S3. Tsix does not affect H3K9 di-methylation within the Xic. ChIP analysis of H3K9 di-methylation in Tsix-truncated (Ma2L, in red) and corresponding control (Ma1L, in black) embryonic stem cell lines. Locations of the primer pairs are depicted in Additional file 1. [file 1756-8935-2-8-S3.pdf]

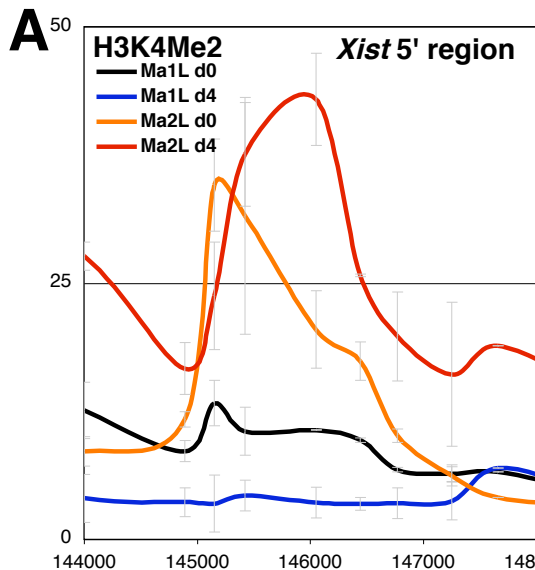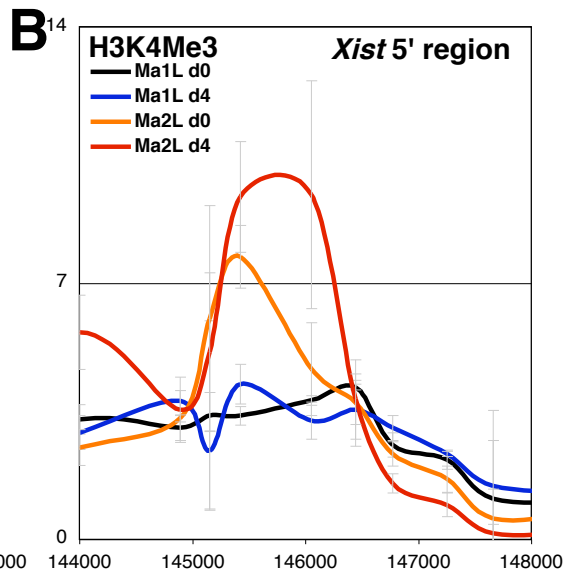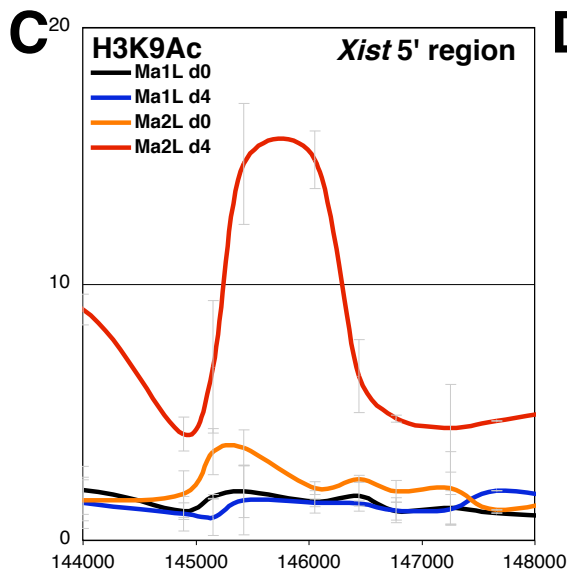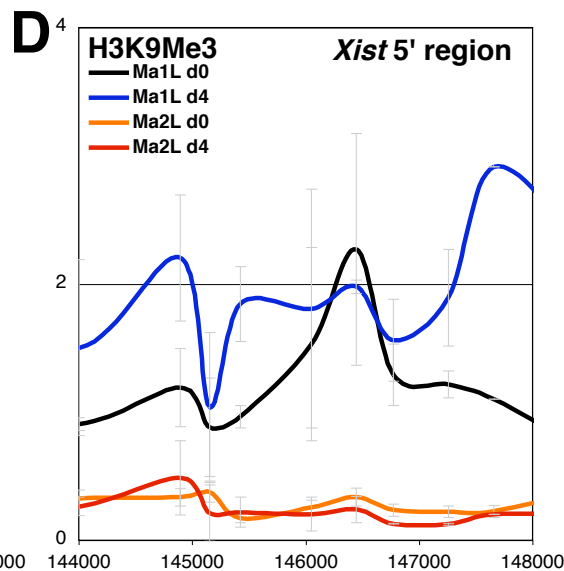

Supplement: Additional file 4 — Figure S4. The Xist promoter region is abnormally enriched in euchromatic marks in Tsix-truncated undifferentiated and differentiating embryonic stem cells. ChIP analysis of H3 modifications around the Xist 5' region in Tsix-truncated cells (Ma2L) and its corresponding control (Ma1L) male ES cells. (A) H3K4 dimethylation (H3K4me2), (B) H3K4 trimethylation (H3K4me3), (C) H3K9 acetylation (H3K9Ac) and (D) H3K9 trimethylation (H3K9me3). ChIP experiments were performed on undifferentiated embryonic stem cells (d0; black and orange lines for Ma1L and Ma2L, respectively) and after 4 days of differentiation with retinoic acid (d4; blue and red lines for Ma1L and Ma2L, respectively). [file 1756-8935-2-8-S4.pdf]

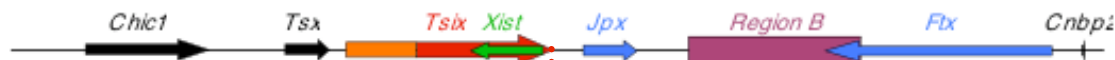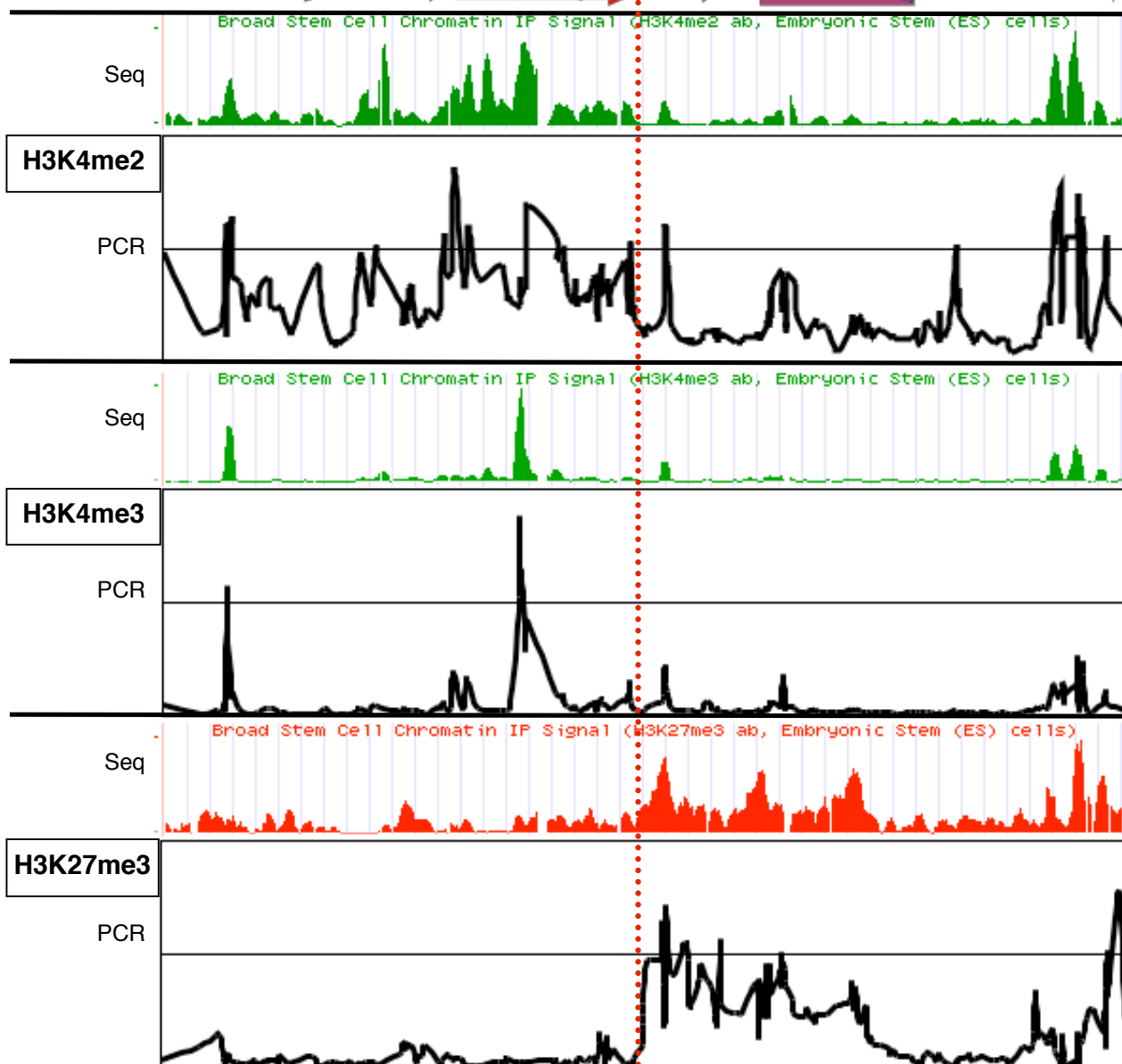

Supplement: Additional file 5 — Figure S5. Comparison of ChIP-PCR and available ChIP-Seq data. A schematic representation of the 300-kb region analyzed in our ChIP experiments is shown at the top. Bottom: results obtained for H3K4me2, H3K4me3 and H3K27me3 from our ChIP-PCR (graphs in black) experiments and from [32] (in colour). [file 1756-8935-2-8-S5.pdf]

*Chic1**Tsx**Tsix**Xist**Jpx**Region B**Ftx**Cnbp2*

60

— H3K27Me3

— H3K9Ac

30

0

0 20000 40000 60000 80000 100000 120000 140000 160000 180000 200000 220000 240000 260000 280000 300000

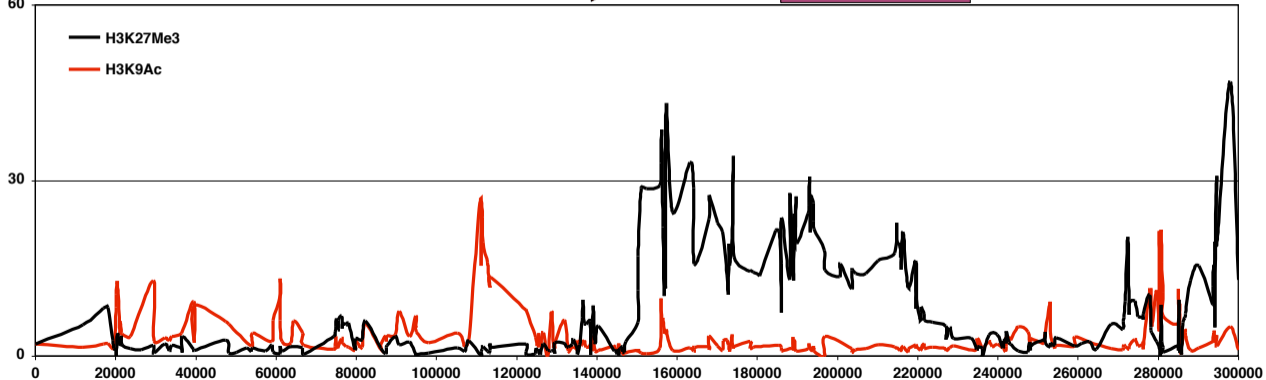

Supplement: Additional file 6 — Figure S6. H3K9 acetylation does not mark the boundary of the H3K27 tri-methylated domain located at the Tsix 3' end. A schematic representation of the 300-kb region analyzed in our ChIP experiments is shown at the top. For legends, see Figure 1A. ChIP analysis of H3K27me3 (in black) and H3K9 acetylation (H3K9Ac, in red) in wild-type male embryonic stem cells (Ma1L). ChIP assays were performed using the set of 383 primers pairs. [file 1756-8935-2-8-S6.pdf]
