# Supplementary material for: A role for non-coding Tsix transcription in partitioning chromatin domains within the mouse X-inactivation centre
Source: Epigenetics Chromatin. 2009 Jul 20;2:8. doi: 10.1186/1756-8935-2-8 (PMC2720958; doi:10.1186/1756-8935-2-8)
Supplement: Additional file 7 — Figure S7. Primers used in the 384-well PCR plate-based analysis. [file 1756-8935-2-8-S7.pdf]

Region COORD	Fwd	Rev
48	CTGAGAGTCCCGGAATGTGT	GTGGAAGCATTTCGTGGGTA
248	CAGCCACTTAAGCAGAGGC	AGCGATCCTGTCCAGATGTC
11822	TCCCAATGATGCTTTGTAACTT	GATAGGATTGATCCTGTGGCA
16873	TCCTCCTGTCGTTCAGTGC	GCAAGCACAGTCAGATCAGC
18101	GCCACTGACAGATATGAACCAG	CAGGTCGGTAGTGACTTGCTC
19282	GGGAATGACATATGCGTACCTT	TGTCTGTTAATTAATGCGGGA
19492	GGAGTTCACCTCTACCGGC	CTGCGTGTTGGGTAGATGG
19987	AGCATCTGCGGAGATATGC	CCTCAATGCCCTTCTCATTC
20086	GAATGAGAAGGGCATTGAGG	CCTATTCAACCTGCGGTTTT
20142	CTCTTAGCAAAGGGCACCTG	CTCTTTCCTCAGGGTCGATG
20397	CTAGTGGAAAACGGCGACTG	TGGACAAATATGTGGGTGGA
20974	AAGTGTGCAAGGACTTCTTATCA	CAAGTATGTGCAATCCAAGCA
21063	TGCTTGGATTGCACATACTTG	TGTGACTTGATAGGAAGGCG
21095	TTTGAATATGGTGTCGTGTTCAT	CAAAGGGACTATGTGAGCCA
21342	TGTTTGTCAATTGTGGAAGGC	GGAAGTTGGCACCTGAACAT
21499	TCACTAGGGAGACCATTATTCAAA	ACGAGACAGCAAGTGTGGTG
23617	TGCCACCTTAACTGTGAAGAA	AATATACACCGGGCAAGCAC
26214	CAATCTTTGACAACCACCAGC	AGACAAAGAGGCACTTGGGA
26400	TTCATAGTTCGCTTATGAAACAGA	GCCTTAATTCTGAGGCTTTCA
29386	AACAAGTTTGATACCGAATTTCC	TTCATTACCAGTGAGTCAAAGCA
29573	CATGACTACCAGCCTCTGGC	CAATTACAGCTACCTGCGCT
32319	TCCAGCTAGAAAGTCAGAGGC	TAGATTCGCGGTTATTCCAA
33310	GTTCCAGTCATCCAAACCG	TCACCTTAAGCTTGCCCCTA
33941	GGATTCTGCGAGGCCTTAG	CCTTTTCATTGCAGGGAATC
36460	GCACCATTGCCTTAACAAGAA	CATGGTTCAAGAACTTCGCA
36504	TTCCAATCAGTCCTGTTGCT	TTGGAAGACTTAGCGAGTAAAGC
36568	TTATGCGAAGTTCTTGAACCA	AGGAAGACGCCTTATCCCAA
36677	TTGGGATAAGGCGTCTTCCT	TCAAGCATGAGTTCACACGG
39510	CTGTGGCCACTACATAGGCA	ATATAAGGGTGGTGGCGTGA
39520	GTGGGTCCAGTCAGTTTTGA	ATATAAGGGTGGTGGCGTGA
39611	TCACGCCACCACCCTTAT	TGCTTCATAAAACACGGGAA
47394	TTGGTGAGTTTCCGACCAGT	AGAGGGATTTTGCCCTCTGT
48284	CCCACTTCCTCACATCCG	TGTCTAATTCCGGGTCCTCA
50119	CCACATACCGTTTATTTGCG	GCAAAATAACTCTTTGGGTGGT
52646	CGACCCGACTGAGGAGTTT	TTGAACACAGCGAAGGAGAC
52743	GACAGTCTCCTTCGCTGTGTT	AGTGCAAAACACAAGCCTCA
53840	GCCATGGCTTATCCTCTTCT	CCAATCAGACGGCTGAAACT
54175	TTCAACTGCAAGTGAATGCC	GCTTGTTCCGTTCACTGAGA
57243	TTCTCGGATCTGCACTTGGT	TCTACCAGGCATTGGGTTCT
58895	CATCCAGATAGAGCTGGCGT	TGGTTCGACAGACATAGCCA
59154	TGCTGCTTCTGTCGTTGTTC	AAAGCCACACGGTTCAACTC
60997	GCCCCGTCCAATTTACAAC	TTTCCACCTAGAGCCAGAGG
61016	CCGGGTGGAGAGTTCCTT	TCAGTTTCCACCTAGAGCCA
61056	AAGCTAAACTTCCTCCCGGT	TTGAGGGTGGAGACGCTAGT
61447	CTTGTCAACCTGCATTATTACTGA	ATCTCAGCAGTTGCCAGGAG
63666	GATGCAAAGCTCATGCGTTA	CAGATTTGCACACCATGTGA
64422	TTGTCCCCTCGTTTGTTACC	GAATAGCATCCCCAACGTGT
66565	AAATACCTATCAACGGGCCTT	CGTGTCAATATTAGAGCCCAAAA
66911	AAAAGGCCCGAGATGAGAA	TGCTAGATCTCAAAGGCTTGAA
74332	CAAGCCGTTAATGTAAGCGA	GTACTTGCCAAGCCCAGAAG
74762	AGGGTACTACAGAAGGCTGTCA	ATGCAGCATATAAGGTGGGC
74847	CCAATTCACAGCCCACCTTA	TTTATGCCTTCTACGGGTGC
75008	GGACAATTCAACCCCTCAAT	CCTGAAGGTTCGTTGTGGTT
75435	CACACCCCAACAACAATGAC	ACTCACATTGGTCGGAGGAC
75518	GTCCTCCGACCAATGTGAG	AGGAGCACGTCAAGTGTGTG
75545	TCGGATACTTTAGAGGCTTGACA	ACTCTTCTCCAAGTGTGGCG
75601	CACACACTTGACGTGCTCCT	CTTCGTTGGGTCCGGTACTA
76361	TTAGCCTGCTGCCACAAAG	GGAATGAACGACAGGGCAT
76444	CAGACCTGTGCACCACCA	GCACAACTGGACTCGATTTTC
76521	CTGGTGCTCATTACAGATCCA	GGCTGCCCTATGTTCTTCCT
76627	ACATAGGGCAGCCCACCT	GTGCTTCCTCATTAGAGGGC
77361	GGACCTACTCCTTCATGCCA	GCCTATCTTCCATGGAGGTCTT
77518	ATTCCCAATACCCGAGGCT	TGTGAATAAAGGCCTGAGGG
78140	AATGTTCCAGTATGAGCCTGC	TGTTGTTCTTGAGTAGGTCCTTCA
79702	TCCAATCTCCAACTAACTATTCCA	GAGCTTAAACTGCTGGGTGC
79900	ACAGATGATGACACCGACGA	TTCTTGTTCACTTGGGTGGA

81407	CAGCAGTACCGATGATGAGG	TGTTTAGAGCTTATCCACCCTTG
82169	CCCCAAAATTTCCTTGGTA	GAACTGTCACCGCTCTTTCTG
86069	CAATCCACAGCCAGTCCTACT	TCGAGACCGTGGGTCTATTC
86095	GAGATCCTACCTCTTAGGCTCTGA	CTCCGTTCTGCCAAGTCCT
87354	AAAGATGTTCACGAGGGAGG	TGGTAGCTGATGGTGACACG
87438	AACTGATTAAGCAGGCAGGC	TGTTTTCGGCTTAGTGTGTAGAGA
87452	CGTGTCACCATCAGCTACCA	GATTTGTTTTCGGCTTAGTGTG
87637	TGAAAATCACCGCAAAACAA	GAAACGCAAAGGTTTCAAGC
89700	TTTCCAGATCACGTCACCAA	ACCTCACTCCAGCAATTTCC
90647	TTACTCAGCCGATGCCGT	AGGCAGCAGTTCCTTGCTAA
93265	CAGCCTATCACTCAGCATTTTG	TCTGAAGTGTTGCAATGTTTAGC
94781	CAGTGACAGGGAGGACCG	GGCATAAATCACCGCATCTT
94790	TGGCAGCATGTAACAATCAGA	GGCATAAATCACCGCATCTT
94879	AAGATGCGGTGATTTATGCC	CTATTGCCCAGACAGAGCCT
95019	CAGGGTTACCTAGACACCAAGTG	TGGAACACCATAGTAGGCCTTAG
98033	TTCTTCGGTAAGAATCTGCCA	CTGATCCCCTTACTCTGCTCA
105245	TCTCACTCAACGGAGCTAGATAAA	TCTTTGGTATGGGACAAGCC
106981	TTGTTACATTCCACCAGACAGA	GGCGATAGAGACAAGGTGCT
107496	GAGTACTCCCCACATGCACA	CAAAACGACTAACCAGTCAACAA
108139	TCAATGGGCCTCCTGAAGT	TCCGGCTTTGCCTATACATC
110843	GCCTCAAGAGCCTTAGGTCC	GCTTGCAGGTACTTTTGGGA
110990	TTCTCACTTTCCGAGATATCCA	GCTGGCTATCACGCTCTTCT
111085	AGAAGAGCGTGATAGCCAGC	CCACCCCAACAAGGACTTC
111129	GGGCTAAGGAGAGGGCAG	TATCCCGCAAGAATTTCAGC
111475	TCCCGAACCGATAGTTAAAATG	CCACCTGTAAGGGACAGACA
111640	ATGGCTCAAATGGATGCCT	TAGTGACCTCCCAGTACCCG
111838	TGGGAGGTCACTAAAATGCC	TGATAGCCCAGATCCTCGAC
112756	GCGTAGAGATGGTTGGCTAAA	CTGCTACAAGTCACGCCCTT
112868	CGTGACTTGTAGCAGGGTCTA	ACACGTGCGGTGAGGTAGTA
112973	CAATACTACCTCACCGCACG	AAGCGGAGGTAGAATCCCAT
113178	CGTGTGAAGTAACCAAGAACGA	AACGGAACGCAGTACCAAAA
113273	ATTTTGGTACTGCGTTCCGT	CCGAAAATACTCCTTGTCCC
113289	CGAGGTACATAATGACCCGAT	CCACCCCGAAAATACTCCTT
113423	TCTCAGTGGTTCGTATCTGAAAA	TTAGTGCCATTGTTTGCTCG
113482	CCCTCCCGGTAGTGTTTATTC	AATCCGCATCAAAACCAAAG
122239	CCCAATCCTTTAGTTGCCAG	ACTGCAAGCGAGTGTGAGTG
122254	TGGTAAGGTTTCATGGGCTT	GGTACTGCAAGCGAGTGTGA
122417	TTGTTGGCTCCTTGTATTAGCA	GAAGTGCTCCAACAAAACCG
124480	TTGGTCTTTCGTTCCTCCAG	TCAAGGTGGCTTGCTATGGT
124746	CAGGATAATGCAAAGTAGCCTAAG	AGTTACAGAAGCCCTCCACG
124843	CGTGGAGGGCTTCTGTAACT	TGCAATGTGAGTCCTCATCC
125093	CCTTGTAGAACAGGCGAACC	GCTCAAACGTTTTCTAGACTGAAG
125327	GAACCGAGGAAATAGAGAACCTC	CATCAAGGTGTAGAGGAGGCA
125417	TGCCTCCTCTACACCTTGATG	AAAGGATGCAACCCAAAGTG
125765	GCTTCAAGCTAGGCCAATGA	GTTTGCGAAACCCAACCA
126635	ACTGCCTTGCACAAATCCAT	TTGAGCGAACACATAGATTGC
127710	AAATTAAGCATTGGCTCATTCC	GATGTATCCGATATTGTTTTGGG
128177	CAAAGCATGTTATTCCTAGAGCA	TGAATACGTAAACCATGAAGCC
128356	CACAGCCTTAGTATTCAGCACAA	GGCACACTGGTCTATTGCCT
128763	TGAAATGCACCAAATTACTCG	CTTTGGGCTTAGGTGAGCAG
129123	GGCTATATACACTGGCTGGATTT	GCTGGATAGTATTGTGCAAGGG
129257	AAACGTATTCCTTTATGGGCAA	ATTCGAAGATGAGACCTGCC
129476	AGGATTGGGTCCTTAGGTCTT	GAATGCTGACAACCCAAAGAA
129552	TCACAGGTGTCCTGTAGAAACA	CAGAAGTTTTGCGACTTGAGA
129709	CCGGACGGAATGATGTATTT	AGGAGCTTCATAGCCCCTTC
131843	CGTGAAAGCCTGTTCATCTTC	CACTAGCATTGCAGTCTTCTGAG
131901	TTAGATGCTCTCCACCCAGC	AAATGACCCGAGGATCAACA
132457	TGGCATGAGTAGGGTAGCAG	ACCTTCGAGGAAGCCATTCT
132811	TAGGCCTGTTGCCCAGTG	AGTGCTGGCGACCTATTCC
133726	TGTTCAGAGTAGCGAGGACTTG	TCACAGGGACAAACAATCCC
134057	CTCCAAATTCATCCCTGGAA	ACCACCAATTGAAAACGCTG
135047	TGGGTCAGGACACTAATTTGG	TGTCCATTAGGAGGTGCCAT
135111	AAGATGGCCTCCAGATGGTT	CCTCCACGGCACTGTAAAAT
135130	ATGGCACCTCCTAATGGACA	GTCCTACATGACCTCCACGG
135700	CCTGCTGCGAAGGAATTTAC	CATCCTTGTCCATAACACTTAACA
136360	CAGCTAAGCATAATGCATGGAG	CCTTGTTGGTCCAGACGACTA
136524	TTTCAACCCCAGTAGACTTCC	CAAAATTTCTTCCGTGAGATGA

136683	GTGTACCCCAGCATAATGGC	GTGCCACCCACTGAAAAGTC
136789	TGACTTTTCAGTGGGTGGC	TGCTCCTCCGTTACATCAGA
136807	CAGAAAGAAACTCGAATGAGAAAA	TCCTGCTTGAACTACTGCTCC
137242	GACCTAAGTACTCGGCGGCT	TGCCAGGATCTAATCATTCCTT
138327	GAATTCGAGGTGTCTCTGTTCA	AGTATTGACTGGGCAAAGCC
138436	GGCTTTGCCCAGTCAATACT	TTGTCCATTGACCACTTTTCTG
138648	AAATCCGGGTCATTGTTGG	CACAATGGCAGTTCTGGATG
138838	ACAGTACCGGGTAGTTTCGG	TCAAGGCTACTGAGTCCCAGA
138994	TTGCATAGTAATCATCCAAATGC	TTACACATATGACCTGTGCGG
139507	TGAAAGGCGAAGGAGTATGG	CCTGCAAGGGATACCGTTTA
140352	GGAGTCCTGATCTAAAAGGCA	TGCATCTGGTGAAGACTTATTTG
143748	GCAAAGCCTGAACTGGTATAGAA	TCCACCGAGCTACTCTTCAAA
144094	ATGCAACCCCAGCAATAGTC	CTTTGGCCAGAGTCATAGTGG
144233	GCTTGAGATCAGTGCTGGCT	AGTGCTCTATACGTGGCGGT
144266	GGCCAAGGTGTAAGTAGACTAGC	GGACATTTGACTCATCCGCT
144324	GAGTTTTAGTTTGCACCGCC	CGATGTAAATCATTGAAACCTCA
144379	GCGGATGAGTCAAATGTCCT	GAAGTGTTGGATGATGTCGG
144486	GATCCGACATCATCCAACACT	AAGGACGTGAGTTTCGCTTG
144732	TTACGTCAAGTGGCAACCAA	CTTTAGCTACTTGGCGGGCT
144908	TCCGCCATCTTAGACACATTC	CTCAAGTTCTTGGTGCGGTA
144990	GCGTTATACCGCACCAAGA	GCTCGACAGCCCAATCTTT
145021	CGCCATCTTTTCCTGTACG	GTCTATAAAATGGCGGCTCG
145216	ACCCACGCTGAGCCCTAT	GATTCGCCTTGATTTGTGGT
145292	TACTTCCGGCTAGCACAACC	AGCGGACTGGATAAAAGCAA
145321	ACCACAAATCAAGGCGAATC	GCCAAGAATTAGGACACCGA
145344	GCAACCCCGCACATATAAAG	ATACGGCTATTCTCGAGCCA
145400	TTTATCCAGTCCGCTGTGCT	AACTGAGTGGGTGTTCAGGG
145421	AATTCTTGGCGTAACTGGCT	TTGCTCTTAAACTGAGTGGGTG
145454	CGAGAATAGCCGTATCACGC	ATCGTTTGGTGCTGTGTGAG
145496	CCTGAACACCCACTCAGTTTAAG	TGCCTCTTATTTGCGTGTACTG
145643	ATATTTCCATCCACCAAGCG	GCCTCGGATACCTGCTGTT
145693	GTATCCACAGCCCCGATG	CCTCGGATACCTGCGTTTAG
146340	ACATCCACGGGAAACGAG	CAATCTTTGTGGCCACTCCT
146446	TGCAATTCTGACATCTTATTGGA	GAAATTCACGCGTCATGTCA
146557	ATTTTGCGCGATAATGAAGG	TGCATAGACAGGTGTGTGACC
146832	AGTTGTGACCGATTTGGTGG	TGCTAGCGAGTAACGTACTTCAG
147131	CAAAGGATATTGTTGTCTCGTTG	TGGCACTTTGATCGTCCATA
150170	GAATGGGTGGACACAGTCAA	CAGAGGTACAGGTCCGGAGT
150233	AACTGGTTCTACCCACGAGC	TAACACCGTTCCTCTGCCAT
150248	CACAACTCCGGACCTGTACC	GGCCTAACACCGTTCCTCT
150283	CAACTGTAGTGCAGGAGTTGGT	ACCTGGCAAGCCATACTCTC
151066	CTGTTCTTCCACTTGCCACA	ACCATCAACTTGCATTGCCT
152369	AATAGGCCACTGGCAGTTTG	TCGTTGAACCCTTCTATGGC
152678	AGCTGCTGGAGGTGTAATGG	GCTTTGGCAGCTTCGAGTAT
152884	TGAGCACCAAATCAACCTGA	AACCACTACGCAACAGCAAC
152930	GGAATGGGTAGGCCTCTGTA	TCCTCGATATCATCCTTGGC
155712	AAATGTGCTACCTTCTGGAGAGA	ATGTTGGAAAGTTGCCGAAG
156036	CCTCGTGGTGCAATAGGTAAA	TCACACCAATACTGGTTACCACA
156190	TGAGGTGTCCAATAGGAATAGG	ACAGAGGCTAGGTGGCAAAG
156493	TTAAAGACGGGCAAGAAACG	CAACGTGACCGGGACTGT
156662	ACAGGGACTGTTCGGCTGT	TAATCCCGGGAAGGACGTAT
156738	ACTTATAAAATGGCGGCGTC	TCACCAGGGCATGTTCATTA
156764	ATACGTCCTTCCCGGGATTA	CTCTCTGACAGCGCGAGAC
157016	ATGAGTGATCAGTGCCCACA	TCCGTCAAACCCCAATTAGA
157029	GACCTCCACGGATGAACCT	TCCGTCAAACCCCAATTAGA
157270	TTTTGCCCTATGCCCTTTT	CAGATCACCCTATAAGGCCC
158862	ATAGCGCCCGAGACTCAAA	GCTGAATCAAAGTCCTGAACC
163184	TGAAGAGCACTGAAGCTCAAA	AATCAAGAGAAACGTTACCAGGTT
163267	GGAAACCTGGTAACGTTTCTCTT	AGTCCAACCCACCTTGACAG
164083	CTGGACCGATAGTTGGGAAG	ATGCCACATAGCCTACAGGG
164224	GAGTGAGCCCCAAACTTGC	AGGCCTCCATGATGTGTGAC
164243	CTCCCGACTTAGCCTCCTCT	CCAGGACAATAAAGGCCTCC
167885	GAGCTCTTGTTCCACGTAACTG	ACCAGCAGATGAGGCACACT
167936	TGCTGTACTGTAGTGCTCCTTAGC	GAGTAGGGAGCAGGAATCCAC
168029	ATTGTGTGGTGGATTCCTGC	ATCTGACCCTGGTGAATGGA
170043	GCAAGATCCGCAGACTCAG	AACTGTAGGGCACTTCACGG
171391	CAGCTAGACCGAAACTTTCCA	ATTCCACATGCAACCAGTCA

171486	TGACTGGTTGCATGTGGAAT	TGTATGGCTTGCGATAAGGA
172427	CCCTTTCTACTCCACGTGCT	CAACTCATTATTGGGTGGCA
172782	AGGGCCTAGAAGCCACTGAG	CTGGTGCGGGTGCTTAAA
172876	TTTAAGCACCCGCACCAG	GATGTGTACTGCAAGACGGC
172929	CCCAATTTGGTAATGTCAATCA	TAATGCTTTAGGCCAGCCAC
172960	GCCGTCTTGCAGTACACATC	CCTTCCCAATGGCACAGTAT
173019	GGCTGGCCTAAAGCATTACC	CTCAGCTACCCCATCATTGAG
173147	AGATAGCTTGCTGCATCCGT	GCACAGTAGCAGTGCTTGAAA
173754	TCCAATCTCCGGTTAATTTTACT	AGTTTGTGATCCATGGGAGG
173770	GGCAAAGGTTGATGGAACAC	AGTTTGTGATCCATGGGAGG
174074	TGGCATGGAAATACCGATCT	CTATACTGAGCCCGTGAGGTG
174091	TCCTCATAGCTTTTGGGTGAG	GTCTATACTGAGCCCGTGAGG
174178	ACCTCACGGGCTCAGTATAGA	CCTGGTCCTTTTGGCAGATA
178119	TTTCGAAATCATCATCACGC	GCTGCCCTCTTTGAAACATC
178149	GCTGCCAGCTGTCAAACA	CCATGAAGCTACCAAGCCAT
178907	TGCTAATGCATACTAGGCTACCAA	ATAGCTGTGCAGGCGAGTTT
180396	GGGCAGCCGTGATTATTCT	CTCTCCCATAGCAGGCAGAC
180522	AAACCCTACTGCCAGATGGA	TCTTTAGCCCATGCTTCACC
184495	GAATGGTAAGTAATCCGGAGGAG	AATCCTGCTGCTATTGTGGC
184731	CAGGAAACGAAGGGACCTG	CCTTACAAGGATAGGTGAAGGAAA
185376	CAGCCCATTATCTACCTGGC	TCTGTTCCCGAGGTTCTGTC
185924	GACATGTGGTCACTCTTACTGGA	GCCTGTTGTATTGCCTTGGT
186034	GCAATACAACAGGCAGCAGT	GAGCATGTGCTCACGTTCAT
186064	ACACATCAGCAAACCCGAG	TGTGTATGCCCTTTGAGCTG
188026	ACCGCAGACAGCAGAACC	GAAGTTGAATGTTGCACCTCA
188112	TGATGAGGTGCAACATTCAA	TGCACCAGAAATCCTAGCGT
188705	TTCTGGTGGTCCCAGACAAT	TGGATCCACAATTACAAAGCA
188733	ATTAAATTCACGCCTCTGCTT	CCAGGCCAAACCTCATAAAA
188751	CATTTTCCTCTAGACCTGCCA	CTTATAGTGCACCAGGCCAA
188837	TTTGGCCTGGTGCACTATAA	ATAGATGCGGCGACCATTAC
188900	TGTTTTACGATGCAGCTTTTACA	GGAAGAGCGTCTCATTCCTCT
189110	GCTAAGTTCGCAGTGGCAAT	GTGACTTAGTGGGCCCAGAG
189220	AGTCACTTTCCATGGCGG	TTCGCTTCGAGGTCTCCTT
189250	CAAACCTGTAGAGGGCAGACA	CCGAGTCTCCTCAACTCACC
189427	GGGTACTGTTCAGGCCCTAAG	CCTTTGCCTAGTGGCTTGTG
189640	GGCCTTTGCCAACCTTTT	CATCTGAACCGCAGCTTTC
189728	GAAAGCTGCGGTTCAGATG	TCCAGTTCAATGCGGATGTA
192652	TCAGTTTCGGTTCTTTAAAGTTTG	TAATCAACTGTGGTGGGCAA
192811	GAGTGGACTTCCTGGGATGA	GTTGGAGTTATGGCCTCGAA
192916	GGCCATAACTCCAACTATACAGAA	TGTGCAGCTGCCAATCTACT
193017	GCAGCTGCACAAGATAGGC	AGGCTGCGTCTTCTTCCTCT
193114	AGAGGAAGAAGACGCAGCCT	CTTCTTTATAGGGCGTTGCG
193127	GCAGAAGAAGTCCTGAAGCAG	CTTCTTTATAGGGCGTTGCG
193209	CGCAACGCCCTATAAAGAAG	ATGCAAATGCTTGCGTGTAG
194023	ATGGAACGCACTGAATGTGA	TATATGGACCCAGACCCTGC
194058	CTTCATGCACCCAGAGTGTG	GGAAGGATGGTCAAATCAGC
194107	TATTGTGCATGCAGGGTCTG	TCTGCTCCTTAGCGAGTTTCT
194159	GCAGCTGATTTGACCATCCT	CCTGATGTATGCCAGGTCTGT
196761	CTTCCCCGCTGCAACTTA	TCAGAGAGCGATTTTGGGAC
196867	GTCCCAAAATCGCTCTCTGA	TGGTGATTTAATGATTTGGTGC
196881	CTGCTGCTAGTGGAGGCTGT	TGGTGATTTAATGATTTGGTGC
200588	CTTGTCCTCAGATTGAGTTAGCA	CAATGGTTGTTGATGCAAAGA
200613	TGACTTGCCAGTTTTGAGGA	ATATCCCGTGACCCCATACA
203508	GGAGGAACCTTTCCCCTGT	TGATGATGGCAGGTAGTAACG
203561	TCCCGAAGAGCCTTTGTATG	GGTAAATTGGCTAGCCCTCA
204448	GCCAGATACTCATCTTAACACACC	GCCTAGCCCTACCTCTAAACC
206341	TGACTTTGCAGGTGTCTTGC	ATTAACAGCCGTGGAACACC
207030	TCTTGGTGCTTCTAGATCTGCT	GAGCCTACGGTGAAAACTACAAA
210140	TTGGACTACTGAGTAAACACCAGG	CAAAGAATTCGACTATGCCCTC
214271	CTGCAACAGACTGTGCTGAC	GGCAGTGCAGGATTGAGTTT
214698	GCTGCAATCTGCCTCCTAGT	GCTGGTTGAGCCAGAGTCTAA
214807	CTGGAGGCCTGCTAGAGACA	GAATTTCCCGCCTGAAGTAA
215514	TGAGGTCAGATGGGTCTCTTTT	TGAAGGAGTCTACTGGGAGCA
215761	TGGAATGCCAAATGAAGTCC	CCTGGTGAGACGGTCAGTTT
215819	ACACCAATCATGTAGGTGGAAG	CCAGGCCTACTCTTCCTCAA
215832	CAAGACATGAACCCTACAGGAA	GGTTCCCAGGCCTACTCTTC
216306	CAGAAACGAACAGATTTATGCTCT	TTCTGGAGGTGGCTAAGG

217547	CAGTGGAGAGTTCCTGCACA	GAGCATAGGCTCAGCCCC
217589	GGAAGACTTTAGCATTCCTCTGA	GTGTTGCTGACTTGGACACC
219464	GGAAGGCTGCTCAAACAACT	AATCTCCTCATGCAACACCC
219477	AATCCTGGACTCGGACCATT	AATCTCCTCATGCAACACCC
219558	GGGTGTTGCATGAGGAGATT	GAGTACAGCTCTTCTGCTCGAC
219722	GGGAAGAAGTTACTTTACCGACA	AAGGTTCGGACCTGAAATCC
220703	GCCTGGGTTACTTACTTGGC	AGGCAAAGCCCCATAGTGTT
220785	GCAAAGTGGGTGAGAGACTTC	CTGCGGCAGGTTTACAGTCT
220840	GCCATCCTGGCATGAGAC	CGAAGAGGTGGCCTAAATCC
220854	GGGCTAGAATGTGCCACAAG	CGAAGAGGTGGCCTAAATCC
221540	CTTCCTGGTTATCACCGCAG	TGATTTACCAGGCTATCGGG
221573	GAAGATGAGGGACAACAGGC	CATATGAGGCTCGAATTGGG
223668	TTGGCTAAATGGATTACGCA	TCAATCTCTGTCAAAGCCACA
227051	AGTGGGCAACTGTCTTAGCG	GCACTGCGATGAAAAGATGA
227063	ATGGTTCAGGCAGCAGGA	TGCACTGCGATGAAAAGATG
227148	ATCTTTTCATCGCAGTGCAG	ATGTCCTTCACGCCTTTCC
228020	GGGAAAATTAGCTAGGGCATC	GACCAAGCGTACTGTTGAAGG
228913	GCAATCTCATGCATTGGTGA	TGCTGGACATAAGGGTCTAGG
233183	TGATCATGTTACGTTCACTGAGG	CCAACACCCAGTTGACAAGA
234664	TGTGGTTACCTCGGACAGTATTT	TGTCTGAACTTCAACGCTGATT
234827	AAGTCGTTTGAGGACGAACTG	AATTTCCATTCGCTGTTTGG
236136	ACAATGTGCACCGGATTAGG	GGTTGTATTATCATTGTCCGAGG
236203	GGATTAGTTTGATAGACGAGAGCC	CAACCTGGAGTGGTGTTCCT
236217	CCTCGGACAATGATAATACAACC	CAACCTGGAGTGGTGTTCCT
236250	GTGCTAGAACACTTAGCAGGTTG	GCCTTGGTGATCAGGAGAAC
236662	CAACACCATTGACCATTCAAA	GGCCTGTTTGTTAATTGCGT
237771	CAGACAATCTGGTATAAATCGTCA	GGACTGCCAGAAAATGGGT
238513	CCTCACTGGAATGCATCAGA	CCTTAACCGCTTTGATGGTC
239850	AAATGATGCCCATACCCTTT	ACCACCAGGCATCTTGGTAA
241320	AACCCTTACAAGATTGGTAGTGC	GGTCAAGTAGGCAACAAATGC
241411	GCATTTGTTGCCTACTTGACC	ATGTGTAAGTGCCCTCCCTG
241945	CTACTGCATTCAGGCTCATCC	GGAGCTCCTGTGCATTAACC
241969	CCCCTCCTGATGACAAGAGA	TGATAATTCCGAGTTTGTGGC
242020	ATTCTAGAGGTTAATGCACAGGAG	TATGCATTCTGCGTTTGACC
242115	AAGGTCAAACGCAGAATGC	TTGCTGTCGTTCCCATTAGA
242683	GAACAGGCTCCAAGCTAAAGAA	AAGTTGCCCAGCTTATCAGG
243197	TGCCTTGTCAACAAACTGGA	TCAGCCTGACTGGTTGATTTT
245000	TCAAGGCACATTACGGAAGTC	CACATGACTCAGCCCAACTG
247326	CTACAAGGTCACTAAACCAAGGAC	AGCTTAGGCATCTGCAGTTTT
247534	GGTGCCTAAAAGTCTGCCTC	TGGATTGCTTAACAGTCAGGTG
247695	GCAGGACCCCATATTATCCA	GAAATTCTTTGGGCTTGTGG
247996	AAAGCCAAGGAACCATCTAGG	AAACTCGGAGACGTAGGGAAA
248075	CCGTGCTTTCCCTACGTCT	CAGTAAAGCTGGCGTCTTGA
248150	GAAATTCAAGACGCCAGCTT	GCCATGTGGCAGGTAAAGA
251455	GGAACAGGACAGGTCTAGCAA	CAAAGCCTCATCTTCGCATT
251660	GCAAAAGCAAATCTGGTACCTAAC	CTATGGCCTGTGCCGTTAAT
251782	CCGGAACTTCTGCCATTCTA	CCTGCCATAGGGAGGTAATG
251797	TGTCTCCAGTCCAAACTACCA	CTGCCATAGGGAGGTAATGG
253041	GCCCTTTAACCTAACAGTCACTAA	TGTCTATTTGTGCTGGAGCC
253080	TATACCCGGAGGGTGTTCTG	TGGAACCCAAGGTCAGAATG
254258	ATCCACGCATCCAGGTAAAC	TATCTTTGGACCTGCCCATC
254531	GGAAATATCCTAGGACCTGACAA	AGCTCTCATCGCTCACCAGT
259063	TCATCAACCACTTACCTACAGGA	CTTTCCGTTCGACTTTCAGG
259147	CAGAATCCTGAAAGTCGAACG	CACCAAATCATGGGTTGTTTC
260921	TTTTCAGCAATCAGACTGTCG	CTGATGTGATTGAGGTCCCC
262817	CAAAACCACCAACTAGTCTTGGA	GTAGCCTCCTATCAAGGCCA
264566	CCTGATTCCCATTAGCAGC	TTGCCCTGAATGCCTATATGT
268196	CCCAGCTTCCGTTCCTTAG	AGCACAAGTCAGTTGACACGA
268630	TGTCGTGAAGAGACAGTGGC	CATTAACACAACGTGGCTGC
271059	CCTATCTTTGAACGGGAGACA	TGCCTTAGCCTTCTATGCAA
271278	TGATTCACAAAACTGGGCTC	TGGAGGTAGGATACTAGCCATTG
271455	ACCCATGGTTCTAAGAAGGC	ATAACAGGTTTGGGTCGCTG
271701	TTCACCACGCACTACCTGTT	TTCGTATCGGTGAGGCTACA
272456	TTTCCATCGAATTCTTCTGTCA	GCAGTGACTTCAACAGAAGTGG
272748	CAGAGTGCCATGCTGAATGT	GCTTTGGATTTGCCCATTAG
272776	CCTTGCTCAACCCCTTGTAA	TGGACTCTATGTCGTGGACCT
274191	AATAATTTCCTACGCGTGAACAG	TTGCAGGGTATATATGCCAAA

274915	GCCTGCCTAGAGTACTAAGCCT	GCACAGCCTACCTAAATACAACC
276128	TCCAGCTGGCTAGCAGAATAA	AGGAGCAGTTGGTGTCCACT
276187	TCCTAACATACCCGCTCTGG	CACACTTCCAGCAGCAATTC
276239	CCTGTGCCACGAATAAATCC	CCTTCAGATGCAGCAACAAA
277744	AAAGCAGTCGGAGCCAAAA	TATAAAGCCATGTAGCCCCG
278071	GCCAAGGCCTAGTTGTGCT	ATAGTGGGCCCATGCATATC
278123	GAAGTTTCCCCATTGACTGC	AGATCTCCGCTGCCAGGTA
278207	CTGGCAGCGGAGATCTTG	ATCTTCTTGCGCTCCTCCTT
279424	GACATTCAATGGTCATGCTAAAA	TGTCTGGTCCTACTTTACGTCC
279595	AGACGTAATTCGCACGCAC	TTGCTCCTGATCCATATACGC
280152	CCCGCGTACTACCTGACACT	AACCATTTTGTTGGCTCCAG
280183	AGAGGCCGTAGACGCGAT	TCTGGCCCTAAAGATCCCTT
280503	AAAGCGTTACTTTCACGTGGT	CATGTCAGATGCTGCCACTA
280743	CCCTGAGCATCCATCAGTAAA	CCATTAAACCACGATAGGGAAA
280874	TTCTCAGGAATGATGGGTTTT	AGAGCACCGGAAAGTGAAGA
281130	CAAGTGTAGAGTCCTCCCTTCAA	TATGGAACAACCATGGAACG
281145	AAATCTGGTAATGATTCTGGACAA	TATGGAACAACCATGGAACG
284925	GTAAGCCCCGGGACACTT	GAGCAAACCACCTCCTGAAA
285019	TTTCAGGAGGTGGTTTGCTC	CACAGCGAAGTGCGTTAAAA
285033	GGTTTCAACCAATGTAGAAGCA	CACAGCGAAGTGCGTTAAAA
285129	TTAACGCACTTCGCTGTGG	CATGTTGCGCTTCTAGTCCA
285140	CAACGTTCTGCTACTCTGGTG	CATGTTGCGCTTCTAGTCCA
285226	GTTGGACTAGAAGCGCAACA	CCAGAATCTTAGTCTCTCCCTGG
285262	AAGCAATCTTTCGCAGCCT	GAGTGGAGGGTGTGACCAAA
285362	GTTTGGTCACACCCTCCACT	CGGTAAGTTGTCCTTCGCTC
285457	CGATTTGAGCGAAGGACAA	GTATCGAGAACTGCGCGTG
285614	CTCTTACGCATGCGCACTT	GTAGGACTCTGCTGGCACG
285660	CATGCGTGATTTCACCTTCT	GTGGCGCTCAAGTACCCTT
285718	CGTGCCAGCAGAGTCCTAC	GTAAAGATAGGCGCGCTTTG
286036	CCAAAGTTATGCCCCAGTGT	ATGCTTTATCGCCTACGACG
286632	TGACACAAGTTCCCTCCTCC	ACTGCCTGCCGTAAGAATTT
286698	CTTTACCTCCTGCTGAAGCG	TGTTCATCGCCGTTAAGTCA
286719	GCAGTTAAAATTCTTACGGCAG	GCAGGTTTCGTACCTGGTGT
286737	AGTGCCTAGAGGGTGGAGG	GCCGCAAGTATAGCAGGTTT
287982	AGGGAGTGTGATGGAAAAGC	CAACTTTGTTCGAGATGTGAGC
290084	GATTGGACCTGCCTAATGACTT	AAGTTTACGGCCATCTGGTG
293650	AAGGCTTTGTGATGTACGTGG	CCTGCCTAGGATGGCTTAGA
293691	CCGTGTGCAAAGCTCATTA	AGATCTTCCGGTGAGCAGG
293812	CACCGTACCCATGAGCAGTA	CCTCTGGTACGACCTCTTGC
293880	GGGAGTGTCCTAAGGGAGGA	CACAGTCCTTGGCGTAGTGA
293892	GAGGGCGAACAGCAAGAG	CACAGTCCTTGGCGTAGTGA
294066	ACCCAGGCTAAACGAGAGAG	GATGTGCCCAAATTCACCA
294118	TCATCTGGCTCGGGACTG	TGGCCATTCTCACCACATC
294167	GAATTTGGGCACATCCAGAA	GGTAGCAGCTGACTTCGCTC
294248	AGCAAGACGAGCGAAGTCAG	TAAAGGATGGCGTTTAAGGG
294526	TCAGATAGGGCACAGCCATT	TCAAAGACGATGTTCCAAGC
294537	GAATTGGACACTAGTGGATCCTT	TCAAAGACGATGTTCCAAGC
297943	TTTGCATCCCTTCAAATCATC	TCTAACCTTGAATGAGCCTTGA
300027	CAAGTGTGGACCCCAGTAGG	GGCAAAGGCCCTCATTACTT
